# Exogenous GDF11, but not GDF8, reduces body weight and improves glucose homeostasis in mice

**DOI:** 10.1038/s41598-020-61443-y

**Published:** 2020-03-12

**Authors:** Ryan G. Walker, Ornella Barrandon, Tommaso Poggioli, Sezin Dagdeviren, Shannon H. Carroll, Melanie J. Mills, Kourtney R. Mendello, Yanet Gomez, Francesco S. Loffredo, James R. Pancoast, Claudio Macias-Trevino, Colin Marts, Katherine B. LeClair, Hye-Lim Noh, Taekyoon Kim, Alexander S. Banks, Jason K. Kim, David E. Cohen, Amy J. Wagers, Douglas A. Melton, Richard T. Lee

**Affiliations:** 1 Department of Stem Cell and Regenerative Biology, Harvard University, Cambridge, MA 02138 USA; 2 Division of Gastroenterology, Hepatology and Endoscopy, Brigham and Women’s Hospital and Harvard Medical School, Boston, MA 02139 USA; 3 Program in Molecular Medicine, University of Massachusetts Medical School, Worcester, MA 01605 USA; 4 Division of Endocrinology, Diabetes and Metabolism, Beth Israel Deaconess Medical Center and Harvard Medical School, Boston, MA USA; 5 Division of Gastroenterology and Hepatology, Weill Cornell Medicine, New York, NY USA; 6 Paul F. Glenn Center for the Biology of Aging, Harvard Medical School, Boston, MA 02115 USA

**Keywords:** Obesity, Transforming growth factor beta

## Abstract

Insulin resistance is associated with aging in mice and humans. We have previously shown that administration of recombinant GDF11 (rGDF11) to aged mice alters aging phenotypes in the brain, skeletal muscle, and heart. While the closely related protein GDF8 has a role in metabolism, limited data are available on the potential metabolic effects of GDF11 or GDF8 in aging. To determine the metabolic effects of these two ligands, we administered rGDF11 or rGDF8 protein to young or aged mice fed a standard chow diet, short-term high-fat diet (HFD), or long-term HFD. Under nearly all of these diet conditions, administration of exogenous rGDF11 reduced body weight by 3–17% and significantly improved glucose tolerance in aged mice fed a chow (~30% vs. saline) or HF (~50% vs. saline) diet and young mice fed a HFD (~30%). On the other hand, exogenous rGDF8 showed signifcantly lesser effect or no effect at all on glucose tolerance compared to rGDF11, consistent with data demonstrating that GFD11 is a more potent signaling ligand than GDF8. Collectively, our results show that administration of exogenous rGDF11, but not rGDF8, can reduce diet-induced weight gain and improve metabolic homeostasis.

## Introduction

Aging is typically associated with impaired glucose tolerance, insulin resistance, and hepatosteatosis in both mice and humans. These metabolic disorders correlate with increased adiposity^1^, as well as an age-related decline in functional pancreatic β-cell mass (reviewed in^2^). Identifying signals that modulate adipose tissue mass, glucose tolerance, insulin sensitivity, and/or functional β-cell mass in aging mammals could provide new targets for treatment of metabolic syndrome and diabetes.

We previously showed that rGDF11 administration to aging mice results in a broad range of beneficial effects on brain^3,4^, skeletal muscle^5^, and cardiac tissues^6,7^. GDF11 is a circulating blood factor which belongs to the larger transforming growth factor β (TGFβ) superfamily of extracellular ligands. New data published from multiple labs indicate that exogenous delivery of rGDF11 to rodent models of hyperglycemia (e.g. fed a high-fat diet HFD) reduced fasting blood glucose levels and improved glucose tolerance. The mechanism(s) behind these metabolic improvements may be due to a significant, yet transient, reduction in body mass^8–10^. In fact, recent data suggests that rGDF11 stimulates secretion of adiponectin and a caloric restriction-like phenotype^10^. On the other hand, others have shown that higher dosages of exogenous rGDF11 or viral overexpression of GDF11 via plasmid or viral vector caused cachexia, premature death, and anorexia^11–15^. It is possible that these conflicting reports are due to drastically different doses and/or methodology used to artifically raise circulating GDF11 levels. Similarly, opposing results have also been reported regarding how GDF8, also known as myostatin, effects energy balance and metabolism^16–21^.

GDF8 is classically described as a potent negative regulator of skeletal muscle mass in addition to playing a role in energy balance and metabolism^16,22,23^. The mature, signaling domains of GDF11 and GDF8 share 90% sequence identity and can signal through similar receptors (reviewed in^24^), suggesting that GDF11 and GDF8 may act similarly to support redundant biological roles. However, despite similarities in the sequence identity and signaling components engaged by these two ligands, our structural and *in vitro* functional analysis comparing GDF11 and GDF8 indicates that GDF11 is ~10-fold more potent than GDF8^25^, a finding that supports the idea that GDF11 and GDF8 may serve more distinct roles than previously thought.

In terms of metabolic regulation, there are conflicting reports for both GDF11 and GDF8 regarding whether or not systemic elevation of either ligand results in a positive or negative metabolic phenotype. Further, it is unknown how or if age may affect the metabolic response elicited by GDF11 or GDF8. Therefore, we sought to investigate the effect of mature GDF11 and GDF8 ligands on glucose homeostasis and energy balance by administering recombinant GDF11 (rGDF11) or rGDF8 proteins to young and aged mice subjected to metabolic stress imposed by a high fat diet (HFD). Here, we present our analysis, which includes an in-depth comparison of the effects of exogenous GDF11 or GDF8 on glucose metabolism and energy balance.

## Results

**Figure 1:**
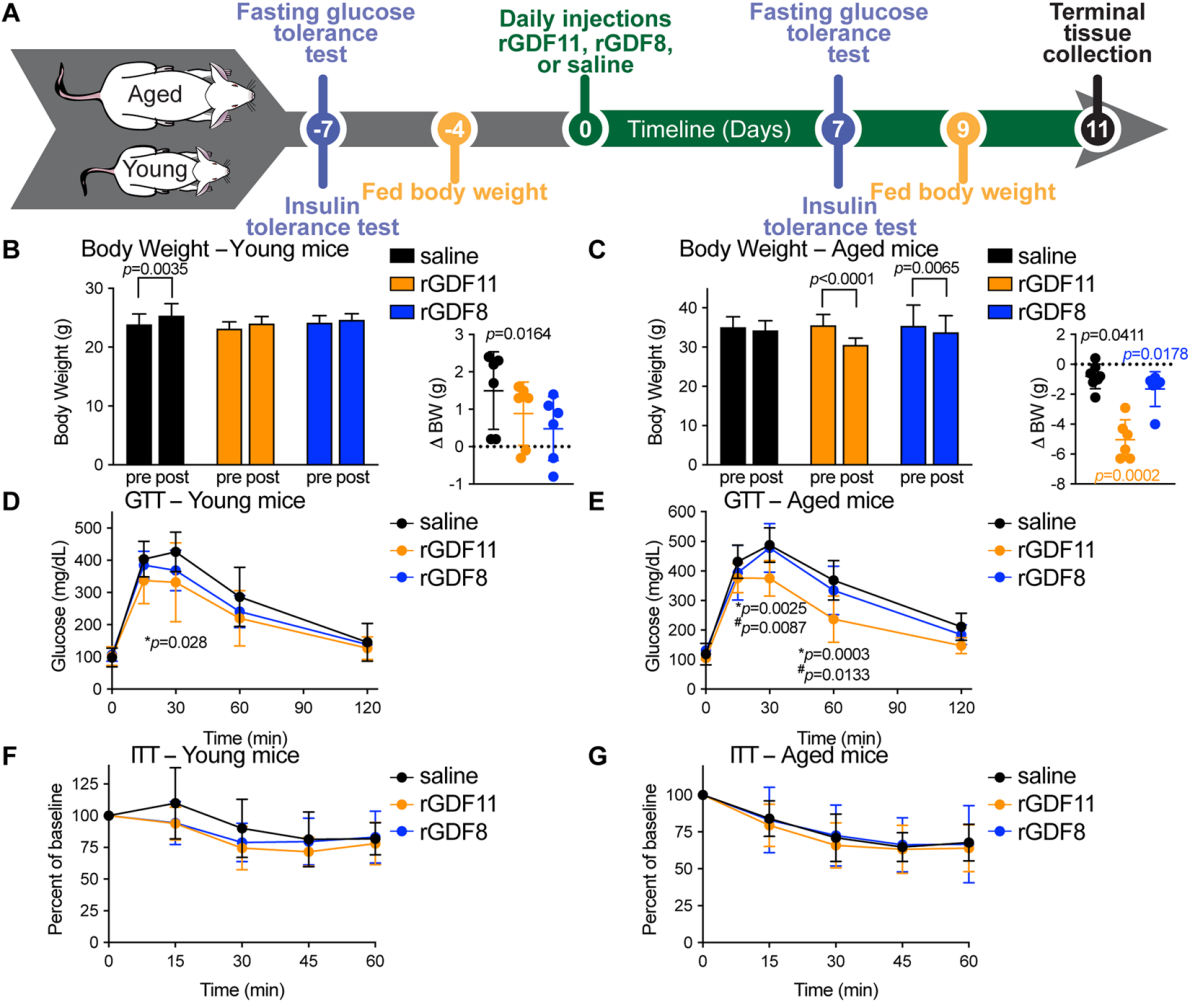
rGDF11, but not rGDF8, improves glucose tolerance in aged mice. (**A**) Schematic representation of treatment schedule and analyses performed on young and aged mice fed a normal chow diet. (**B,C**) Fed body weights and relative weight loss (inset) in young (**B**, n = 6 mice/treatment) and aged (**C**, n = 6–8 mice/treatment) mice were measured prior to treatment (pre) and after (post) 9 days (D9) of treatment with saline (black), rGDF11 (orange) or rGDF8 (blue). Inset: the color of the p-value corresponds the to the group compared (black: saline; orange: rGDF11; blue: rGDF8). (**D**–**G**) Glucose tolerance tests (**D,E**; GTT) and insulin tolerance tests (**F,G**; ITT) were performed on young (**D**, n = 6 mice/treatment; **F**, n = 9 mice/treatment) and aged (**E**, n = 6–8 mice/treatment; **G**, 9–10 mice/treatment) mice. For GTT, mice were fasted for 18 hours overnight before a bolus of glucose was administered (2 g/kg). For ITT, mice were fasted for 5 hours before injected with insulin (young mice- 0.5 units/kg insulin; aged mice- 0.75 units/kg insulin). Data information: In (**B**–**G**), data are presented as mean ± standard deviation. For (**B,C**), 2-way ANOVA with either Sidak’s or Tukey’s *post hoc* test was used for comparison between pre *vs*. post treatment or comparison between groups, respectively. For the insets, an unpaired Student’s *t*-test was performed for comparison between pre *vs*. post treatment. For (**D**–**G**), 2-way ANOVA with Tukey’s *post hoc* test was used. For all graphs: * - saline *vs*. rGDF11; ^#^ - rGDF11 *vs*. rGDF8.

Given the steady decline in metabolic function that typically ocurs with mammalian aging, we aimed to test if exogenous administration of rGDF11 to aged mice could improve their metabolic profile. Our experimental design is shown in Fig. 1A, and employs a dosing regimen that is based on our previous dose-response studies showing that daily administration of exogneous rGDF11 at a dose of 0.5 mg/kg to aged mice is sufficient to reduce age-related left ventricular cardiac hypertrophy in as little as 9 days^7^. Additionally, this experimental design allowed us to measure multiple metabolic parameters on the same cohort of mice before and after treatment by spacing the analyses over the course of 18 days (11 days of ligand administration and 7 days preceding ligand administration) and allowing the mice to recover between the various metabolic tests. Importantly, we also performed a head-to-head comparison between the phenotypic response observed after delivery of rGDF11 or delivery of rGDF8 in order to determine if a ligand-specific phenotypic outcome occurs.

### Administration of rGDF11, but not rGDF8, improved glucose tolerance in aged mice

We found that daily injection of rGDF11 or rGDF8 at equal doses prevented weight gain in wild-type C57BL/6 young male mice (8-week-old; Fig. 1B and Fig. S1), and caused a significant reduction in body weight in aged mice (24-month-old; Fig. 1C and Fig. S1), compared to saline controls. We next sought to determine the effect of each ligand on glucose metabolism in young or aged mice. In order to establish baseline metabolic parameters, we performed glucose tolerance tests (GTTs) and insulin tolerance tests (ITTs) on young and aged mice prior to administration of recombinant protein (Fig. S1A–D). Mice then received daily injections of either saline, rGDF11, or rGDF8 followed by repeated GTTs and ITTs following 7 total daily injections (Fig. 1A). Young and aged mice that received rGDF11 showed a small improvement in glucose tolerance (Fig. 1D,E) but showed little improvement in ITT performance (Fig. 1F,G). However, we observed significant differences in the absolute glucose levels during the course of ITT in both young and aged mice following rGDF11 administration, although these differences are likely due to significant differences in their baseline blood glucose levels following the 5 hour fast (Fig. S1G,H). The metabolic improvements observed in aged mice were also commensurate with a significant reduction in body weight which may explain the improved metabolic performance (Fig. 1C), although no weight loss was observed in young mice (Fig. 1B). In contrast, there was little to no effect on glucose tolerance or insulin sensitivity in young or aged mice that received rGDF8 (Fig. 1; Fig. S1G,H). Together, these data establish the impact of exogenous administration of rGDF11 in comparison to rGDF8 on glucose metabolism in the context of aging mice fed a normal chow diet.

### Improved glucose tolerance in young and aged mice on a HFD following exogenous administration of rGDF11, but not rGDF8

**Figure 2:**
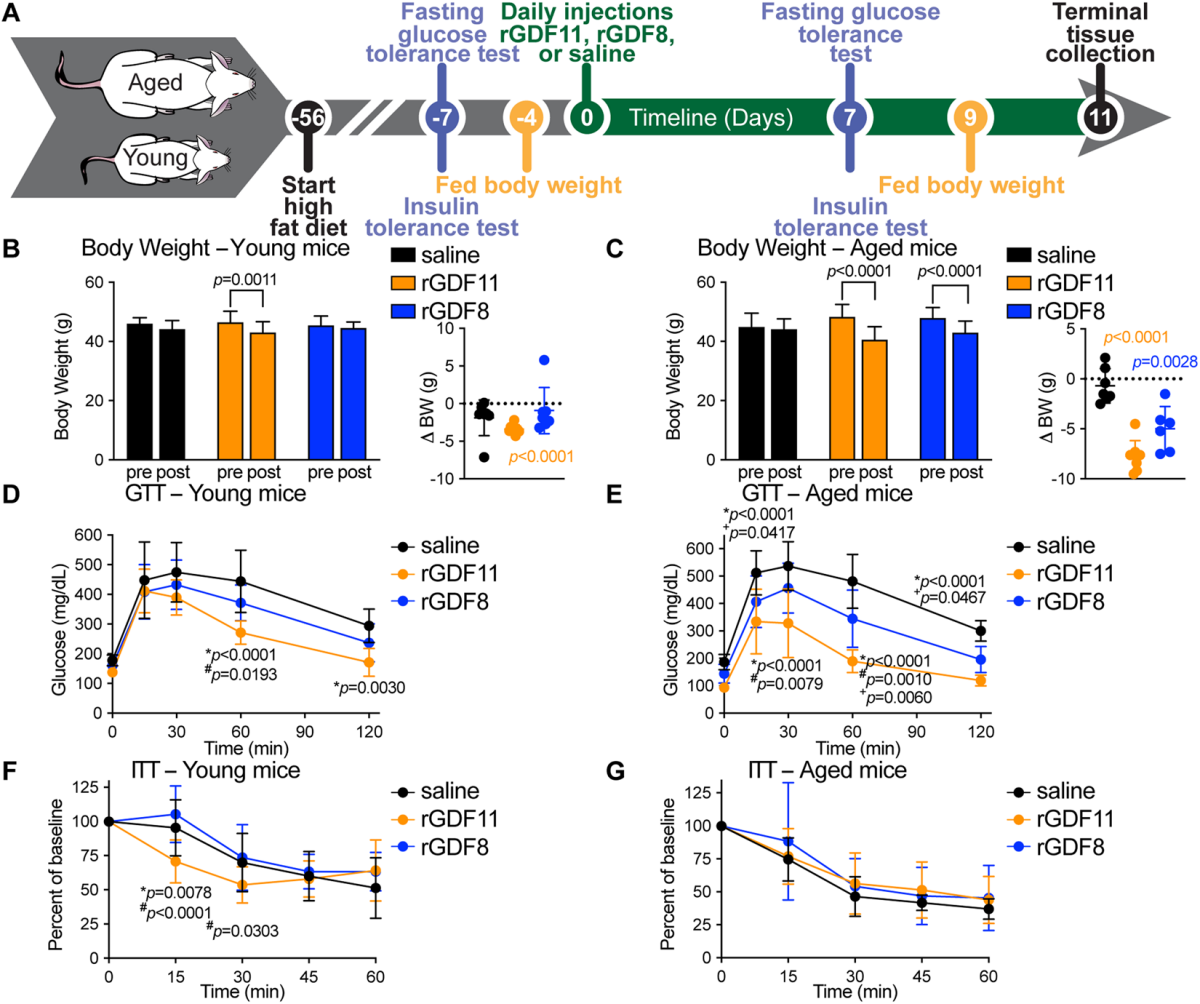
Exogenous administration of rGDF11 and rGDF8 to mice fed a HFD results in reduced body weight and significantly improved GTT performance. (**A**) Schematic representation of treatment schedule and analyses performed on young and aged mice fed a high fat diet. (**B,C**) Fed body weights and relative weight loss (inset) in young (**B**, n = 7–8 mice/treatment) and aged (**C**, n = 6–8 mice/treatment) mice were measured prior to treatment (pre) and after (post) 9 days (D9) of treatment with saline (black), rGDF11 (orange) or rGDF8 (blue). Inset: the color of the p-value corresponds the to the group compared (black: saline; orange: rGDF11; blue: rGDF8). (**D–G**) Glucose tolerance tests (**D,E**; GTT) and insulin tolerance tests (**F,G**; ITT) were performed on young (**D**, n = 7–8 mice/treatment; **F**, n = 8–9 mice/treatment) and aged (**E**, n = 6–8 mice/treatment; **G**, n = 7–10 mice/treatment) mice. For GTT, mice were fasted for 18 hours overnight before a bolus of glucose was administered (2 g/kg). For ITT, mice were fasted for 5 hours before injected with 1.0 units/kg of insulin. Data information: In (**B**–**G**), data are presented as mean ± standard deviation. For (**B, C**), 2-way ANOVA with either Sidak’s or Tukey’s *post hoc* test was used for comparison between pre *vs*. post treatment or comparison between groups, respectively. For the insets, an unpaired Student’s *t*-test was performed for comparison between pre *vs*. post treatment. For (**D**–**G**), 2-way ANOVA with Tukey’s *post hoc* test was used. For all graphs: * - saline *vs*. rGDF11; ^#^ - rGDF11 *vs*. rGDF8; ^+^ - saline *vs*. rGDF8.

Using a similar experimental approach as above, we next asked if exogenous delivery of rGDF11 would improve glucose metabolism in young or aged mice fed a long-term high fat diet (HFD; Fig. 2A). As expected, exposure to HFD resulted in an impaired metabolic phenotype in cohorts of young mice (Fig. S2A,B) and aged mice (Fig. S2C,D). These mice, which remained on HFD, were injected daily with either saline, rGDF11 (0.5 mg/kg), or rGDF8 (0.5 mg/kg) again for a total of 11 days. We found that the body weights of both young and aged mice that received rGDF11 were significantly lower at the conclusion of the experiment compared to body weights at the time that the ligand injections began (Fig. 2B,C). Aged mice that received rGDF8 showed a similar reduction in body weight; however, no difference in body weight was observed in the rGDF8-injected young cohort of mice (Fig. 2B,C). Both young and aged mice that received rGDF11 showed significant improvement in the GTT (Fig. 2D,E and Fig. S2E,F), but only young mice showed improved ITT compared to mice that received saline or rGDF8 (Fig. 2F,G and Fig. S2G,H). Despite the reduction in body weight, aged mice that received rGDF8 did not show an overall improvement in GTT or ITT performance (Fig. 2E,G; Fig. S2F,H). However, our GTT results indicate that aged mice injected with rGDF8 have significantly lower blood glucose levels at some timepoints suggesting that injection of rGDF8 may result in an intermediary effect on glucose homeostasis compared to the effects of rGDF11 (Fig. 2E), despite showing a similar reduction in body weight as aged mice that received rGDF11 (Fig. 2C).

### Exogeneous rGDF11 or rGDF8 does not increase insulin secretion, pancreatic β-cell replication, or promote cachexia in aged mice fed a HFD

**Figure 3:**
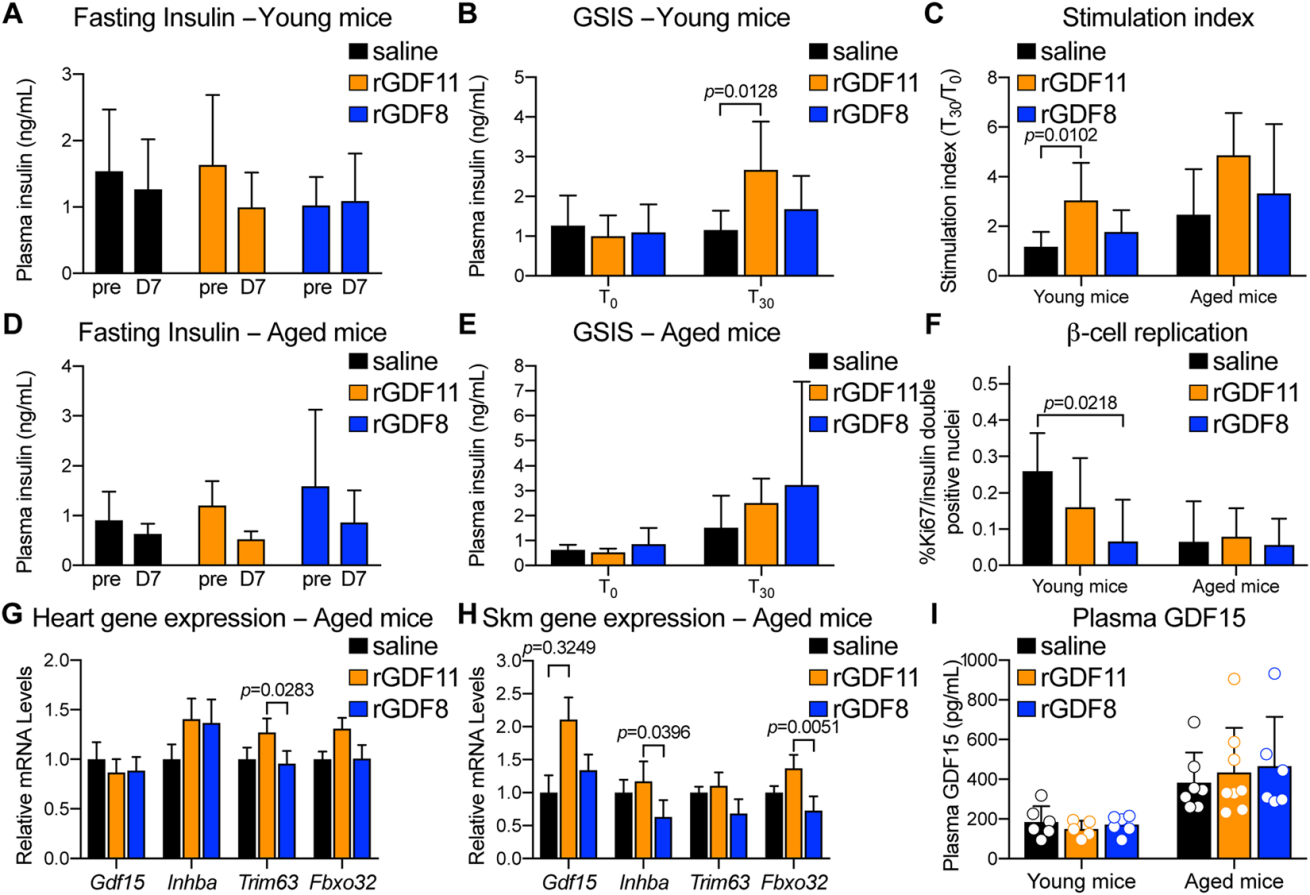
Exogenous rGDF11 or rGDF8 has little effect on fasting insulin levels or glucose stimulated insulin secretion (GSIS) in young or aged mice fed a HFD. (**A,B**) Fasting plasma insulin levels (**A**) and GSIS (**B**) in young mice fed a HFD (n = 7–8 mice/treatment). (**C**) Calculated stimulation index for young (n = 7–8 mice/treatment) and aged (n = 6–8 mice/treatment) mice fed a HFD. (**D,E**) Fasting plasma insulin levels (**D**) and GSIS (**E**) in aged mice fed a HFD (n = 6–8 mice/treatment). (**F**) Quantification of pancreatic β-cell replication in young (n = 4–6 mice/treatment) and aged (n = 3–6 mice/treatment) mice fed a HFD. (**G,H**) Gene expression analysis on the hearts (**G**) and skeletal muscle (**H**) from aged (n = 6–8 mice/treatment) mice fed a HFD. (**I**) Plasma GDF15 levels in young (n = 5–6 mice/treatment) and aged (n = 6–8 mice/treatment) mice fed a HFD. Individual values (open circles) for each mouse/treatment are superimposed on the bar graph. Data information: In (**A**–**I**), data are presented as mean ± standard deviation. For (**A,D,F**), 2-way ANOVA with either Sidak’s or Tukey’s *post hoc* test was used for comparison between pre *vs*. post treatment or comparison between groups, respectively. For (**B,C,E,G**–**I**), 1-way ANOVA with Tukey’s *post hoc* test was used.

Increased insulin secretion could account for the observed enhancements in glucose tolerance, and recent reports suggest that supplementation with rGDF11 or AAV-mediated GDF11 overexpression promotes higher plasma insulin and prevents loss of pancreatic β-cells in diabetic mice^8,9^. We therefore measured blood insulin levels during the GTTs and found no signifcant difference in fasting insulin levels in young or aged mice following injection of saline, rGDF11, or rGDF8 (Fig. 3A,D). Furthermore, neither rGDF11 nor rGDF8 affected glucose stimulated insulin secretion in aged mice fed a HFD (Fig. 3C,E). However, rGDF11 increased glucose stimulated insulin secretion in young mice fed a HFD (Fig. 3B,C). We also measured insulin c-peptide in these cohorts (Fig. S3). We found that young normal chow diet mice, which received rGDF11 or rGDF8 had sigificnatly reduced insulin c-peptide levels in their fed, but not fasted state (Fig. S3A,B). Further, young HFD mice that received rGDF11 showed significantly reduced insulin c-peptide in their fed, but not fasted state, compared to mice that received saline (Fig. S3A,B). We did not observe any differences in insulin c-peptide levels among different treatment groups in the aged chorts (Fig. S3C,D).

It is well-described that TGFβ signaling, including GDF11-specific signaling, participates in pancreatic development and regulation of endocrine function^26–28^. Therefore, we next determined whether the improved glucose tolerance observed after 7 days of rGDF11 administration was associated with an increase in β-cell replication in young or aged mice. Pancreata were isolated from young or aged mice and processed for histological analysis. Replication rates were determined by Ki67, insulin, and Nkx6.1 immunofluorescence (Fig. 3F). Neither rGDF11 nor rGDF8 enhanced β-cell replication in young or aged mice (Fig. 3F). However, in young mice, rGDF8 lowered β-cell replication rates (Fig. 3F). Thus, rGDF11, and to a lesser extent rGDF8, enhanced glucose tolerance in aged mice on a HFD, without concomitant increases in insulin secretion or β-cell replication.

A recent report suggested that very high levels or supraphysiological overexpression of GDF11 promotes activin A mediated cachexia and directly invokes an increase in circulating levels of the TGFβ ligand, GDF15^14^, also known macrophage inhibitory cytokine-1 (MIC1). This study further suggested that elevated GDF15 acts as an appetite suppressant, resulting in weight loss^14^. As it is known that a reduction in body weight can have a positive influence on glucose homeostasis (reviewed in^29^), we asked if exogenous administration of either rGDF11 or rGDF8 resulted in upregulation of the TGFβ ligands *Gdf15* and *Inhba* (activin A), or of markers of cachexia, *Trim63* and *Fbxo32*, in the heart and skeletal muscle of aged mice on a long-term HFD (Fig. 3G,H). We found no differences in the mRNA levels of *Gdf15, Inhba, Trim63*, or *Fbxo32* in the heart or skeletal muscle among aged mice that received saline, rGDF11, or rGDF8 (Fig. 3G,H). Furthermore, we evaluated the plasma of young and aged mice fed a long-term HFD following administration of either saline, rGDF11, or rGDF8 and found that there was no difference in the levels of GDF15 within the age groups (Fig. 3I). Though it is possible that exogenous rGDF11 affected GDF15 expression levels at earlier timepoints, our data indicate that GDF15 expression is unaffected following injection of rGDF11 for 11 days and that the exogenous dosing regimen of either rGDF11 or rGDF8 utilized in this study does not promote GDF15-mediated anorexia/weight loss or cachexia at this timepoint.The underlying mechanism responsbile for improved glucose metabolism following rGDF11 administration remains unknown.

### Neither rGDF11 nor rGDF8 improves HFD- or age-induced hepatosteatosis

Hepatosteatosis is associated with aging and obesity, as well as with metabolic disorders such as diabetes, hypertension, and dyslipidemia. Defects in lipid metabolism and insulin resistance may contribute to hepatosteatosis (reviewed in^30^). Given that injection of rGDF11 prevented weight gain, as well as increased hepatic insulin action in aged mice concomitantly fed a HFD, we sought to determine whether rGDF11 could affect hepatic lipid accumulation. After 11 days, the livers from young and aged mice fed a normal chow diet were harvested and processed for histological analysis. We compared the liver histology from the young and aged mice injected with either saline, rGDF11, or rGDF8 from our normal chow diet and long-term HFD experiments. We did not see any differences within treatment groups in young or aged animals injected with saline, rGDF11, or rGDF8 (Fig. S4) suggesting that this duration of rGDF11 administration does not promote reversal of HFD- or age-induced hepatosteatosis.

### Exogenous rGDF11, but not rGDF8, prevents weight gain in young and aged mice fed a short-term HFD

**Figure 4:**
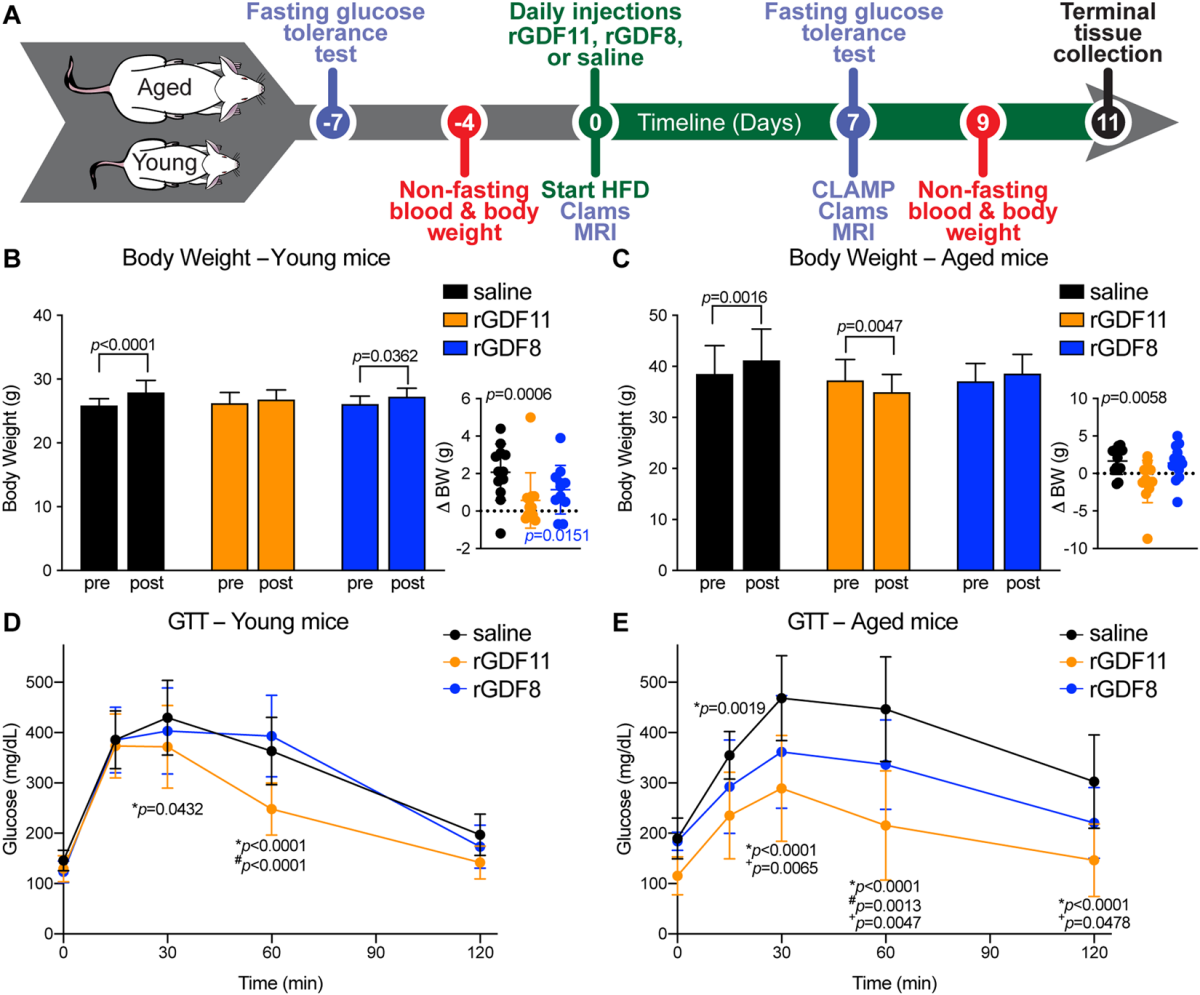
Exogenous rGDF11, but not rGDF8, is protective of HFD-induced weight gain and HFD-induced glucose intolerance. (**A)** Schematic representation of treatment schedule and analyses performed on young and aged mice. Note that ligand delivery and initiation of HFD occur on the same day. (**B,C**) Fed body weights and relative weight loss (inset) in young (**B**, n = 11–12 mice/treatment) and aged (**C**, n = 11–12 mice/treatment) mice were measured prior to treatment (pre) and after 9 days (D9) of treatment with saline (black), rGDF11 (orange) or rGDF8 (blue). Inset: the color of the p-value corresponds the to the group compared (black: saline; orange: rGDF11; blue: rGDF8). (**D,E**) Glucose tolerance tests (GTT) were performed on young (**D**, n = 11–12 mice/treatment) and aged (**E**, n = 11–12 mice/treatment) mice. For GTT, mice were fasted for 18 hours overnight before a bolus of glucose was administered (2 g/kg). Data information: In (**B–E**), data are presented as mean ± standard deviation. For (**B,C**), 2-way ANOVA with either Sidak’s or Tukey’s *post hoc* test was used for comparison between pre *vs*. post treatment or comparison between groups, respectively. For the insets, an unpaired Student’s *t*-test was performed for comparison between pre *vs*. post treatment. For (**D,E**), 2-way ANOVA with Tukey’s *post hoc* test was used. For all graphs: * - saline *vs*. rGDF11; ^#^ - rGDF11 *vs*. rGDF8; ^+^ - saline *vs*. rGDF8.

Our results provided evidence that exogeneous adminstration of rGDF11 to aged mice promotes an improvement in glucose metabolism. We next asked if supplementation with rGDF11 can provide a ‘protective’ benefit from HFD-induced metabolic stress. To address this question, we exogneously administered saline, rGDF11, or rGDF8 starting the same day as the mice were put on a HFD (Fig. 4A). Normal chow diet fed mice were weighed in order to assess their baseline weight and to match treatment groups with respect to body weights and glucose tolerance. Mice were subsequently fed a HFD and injected daily with either saline, rGDF11, or rGDF8. We measured the body weight of young and aged mice after 9 days of either saline, rGDF11, or rGDF8. As predicted, we found that saline-treated young and aged mice gained weight after 9 days on a HFD (Fig. 4A,B). We found that both rGDF11 and rGDF8 prevented HFD-induced weight gain in aged mice (Fig. 4C). In young mice under the same conditions, only rGDF11 was able to prevent HFD-induced weight gain (Fig. 4B). Cumulatively, these results suggest that while both rGDF11 and rGDF8 can prevent HFD-induced weight gain in aged mice, only rGDF11 treatment can prevent weight gain in young mice on a HFD at the dose and time point evaluated here.

### Exogenous rGDF11, but not rGDF8, protects against HFD-induced glucose intolerance in young and aged mice

To determine if rGDF11 or rGDF8 is protective of HFD-induced metabolic stress, we performed GTTs on young and aged mice that received daily injections of either saline, rGDF11, or rGDF8 starting the same day that the mice were put on HFD (Fig. 4A). First, GTTs were performed on young and aged mice fed normal chow diet prior to injection of the recombinant protein and diet change in order to determine the baseline and to match the treatment groups with respect to glucose tolerance (Fig. 4A; Fig. S5A,B). Mice were subsequently fed a HFD and injected daily with either saline, rGDF11, or rGDF8. After 1 week, GTTs were repeated to assess the effects on glucose tolerance. Aged mice, and to a lesser extent, young mice that received rGDF11 showed significantly enhanced glucose clearance compared to saline and rGDF8 treated animals (Fig. 4D,E; Fig. S5C,D). Aged mice that received rGDF8 showed an intermediate phenotype with enhanced glucose tolerance compared to saline controls, but not to the same degree as compared to aged mice that received rGDF11 (Fig. 4E).

In addition to the effects on glucose tolerance, we found that 1 week of daily rGDF11 injections prevented HFD-induced fasting hyperglycemia in aged mice, but not in young mice (Fig. S6A,B). In contrast, fasting blood glucose levels rose in aged mice that received saline or rGDF8 after 1 week on a HFD (Fig. S6B). Furthermore, 9 days of daily injection of rGDF11, but not rGDF8, resulted in reduced fed blood glucose levels in both aging and young mice fed a HFD (Fig. S6C,D).

### rGDF11, but not rGDF8, reduces food intake

**Figure 5:**
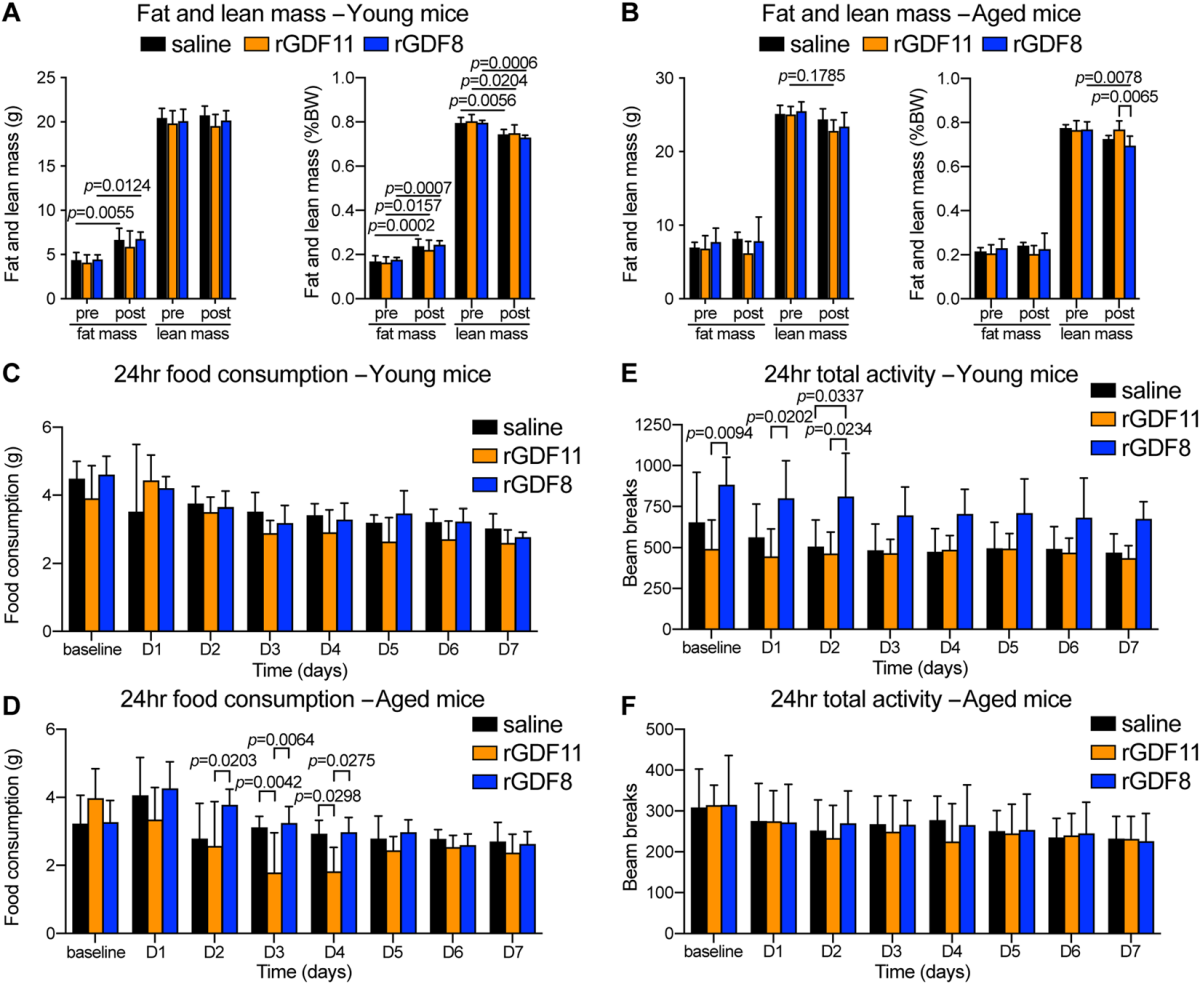
Aged mice fed a short-term HFD with concomitant administration of exogenous rGDF11, but not rGDF8, show a transient reduction in food intake. (**A,B**) Body composition of young (**A**, n = 4–6 mice/treatment) and aged (**B**, n = 5–6 mice/treatment) mice before (pre) and 7 days after (post) concomittant HFD feeding and administration of saline (black), rGDF11 (orange), or rGDF8 (blue). Fat and lean mass values shown on the left and percent of body weight (BW) shown on the right. (**C–F**) Total daily food intake (**C,D**) and activity (**E,F**) were measured from young (**C,E**, n = 4–6 mice/treatment) and aged (**D,F**, n = 5–6 mice/treatment) mice before (pre) and 7 days after (post) concomittant HFD feeding and administration of saline (black), rGDF11 (orange), or rGDF8 (blue). Data information: In (**A–F**), data are presented as mean ± standard deviation. For (**A–F**), 2-way ANOVA with Tukey’s *post hoc* test was used for all comparisons.

Next, we aimed to determine the underlying mechanism behind the altered energy balance in rGDF11 supplemented mice. We performed metabolic cage analysis to measure food intake, energy expenditure, respiratory exchange ratio (RER), and physical activity in young and aged mice injected for 7 days with either saline, rGDF11, or rGDF8, starting the same day as the mice were put on HFD (Fig. 4A). Changes in body composition (whole body fat and lean mass) were assessed using ^1^H-MRS. While young mice injected with saline or rGDF8 gained weight on a HFD, young mice injected with rGDF11 and aged mice from all three cohorts failed to gain weight on the HFD in the CLAMS cages. Indeed, aged mice injected with rGDF11 lost weight during the experimental period (Fig. S7). Young mice, but not aged mice, which received saline or rGDF8 showed a significant increase in total fat mass and no change in total lean mass (Fig. 5A). This resulted in an overall significant increase body fat percentage in young mice treated with saline or rGDF8 (Fig. 5A). On the other hand, fat and lean mass did not change in young mice treated with rGDF11 (Fig. 5A). Interestingly, we did not observe any differences in actual fat or lean mass of aged mice treated with saline, rGDF11, or rGDF8, although aged mice treated with rGDF8 did show a significant reduction in lean mass percentage (Fig. 5B). Taken together, our data suggest that daily injection of rGDF11, but not rGDF8, helps to prevent HFD-induced fat accumulation in young, but not aged, mice and that rGDF11 treatment helps to prevent a loss of relative lean mass in aged mice.

Interestingly, we found that aged, but not young, mice injected with rGDF11, but not with rGDF8, showed a significant but transient decline in food intake as early as 3 days after initiation of recombinant protein injections (Fig. 5C,D). The decline in food intake persisted for 48 hours and the rGDF11 injected mice recovered a rate of food intake similar to that observed in the saline injected cohort by day 5 of treatment (Fig. 5D). This decline in food intake in rGDF11 treated mice was associated with a corresponding decline in respiratory exchange rate (RER; Fig. S8). Upon switching the mice from chow diet to HFD we observed a decline in RER in all three cohorts consistent with the diet change and the animals shifting from utilizing carbohydrates as fuel to utilizing fatty acids as fuel (Fig. S8A,B). However, rGDF11-injected mice exhibited reduced RER compared to saline-injected or rGDF8-injected mice at experimental day 3 (Fig. S8B) suggesting that supplementation with rGDF11 results in aged mice using their fat stores as fuel in response to a decline in food intake. Although analysis of energy expenditure showed a baseline difference between rGDF11- and saline-injected aged mice prior to treatment, the energy expenditures measured from rGDF11- or saline-injected mice showed little difference for the remainder of the experiment in both young and aged mice (Fig. S8C,D).

Reductions in food intake could indicate that rGDF11-injected mice were experiencing stress due to the experimental conditions despite our data showing that both young and aged mice injected with rGDF11 exhibited no change in total activity over the course of treatment (Fig. 5E,F). Based on the stability of activity patterns in rGDF11-injected mice, we believe it is unlikely that their transient behavioral change was due to illness, although we cannot rule out the possibility that administration of rGDF11 induced nausea leading to reduced food intake^14^. Taken together, the data presented here suggest that supplementation with rGDF11 is potentially protective from HFD-induced fat mass accumulation in part by transiently reducing food intake in aging mice leading to the mice utilizing their adipose stores as fuel. Overall, these measurements do not explain the primary tissue compartment or change in behavior responsible for the significant decrease in total body weight observed in aged mice that received rGDF11, suggesting that the weight loss may reflect a combination of changes occurring in multiple tissue depots. Interestingly, young mice injected with saline, rGDF11, or rGDF8 did not show differences in food intake, RER or total activity (Fig. 5E,F and Fig. S8), which may be expected since these mice were young and healthy when the experiment commenced.

### rGDF11 increases hepatic insulin action in HFD-fed aged mice

**Figure 6:**
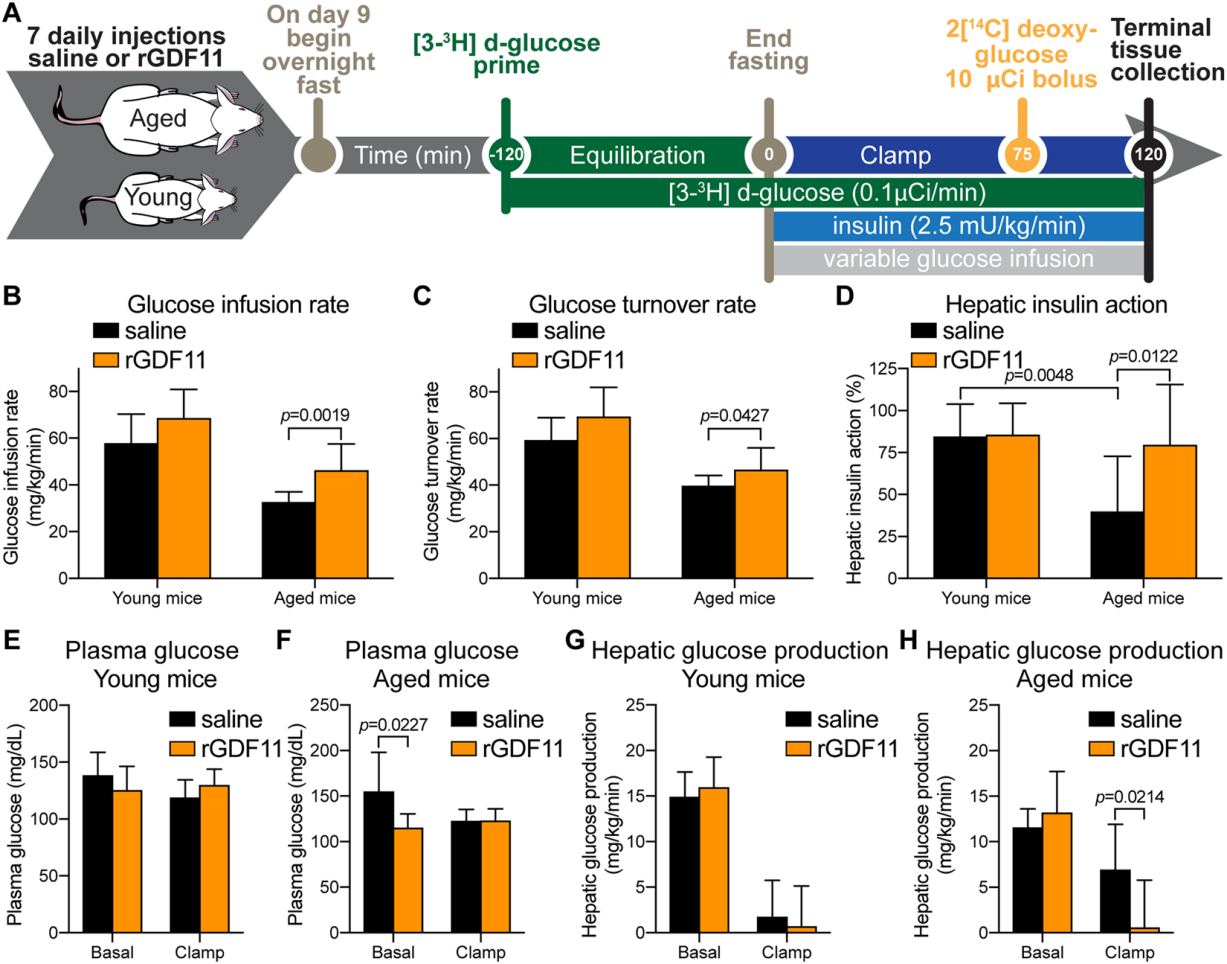
Aged mice fed a short-term HFD with concomitant administration of exogenous rGDF11 show increased insulin action. (**A**) Protocol design of the hyperinsulinemic-euglycemic clamp experiment following 7 days of exogenous administration of saline (black) or rGDF11 (orange) to young or aged mice. (**B,C)** Glucose infusion rates (**B**) and glucose turnover rates (**C**) for young (n = 6–7 mice/treatment) and aged (n = 7–10 mice/treatment) mice. (**D**) Calcuated hepatic insulin action for young (n = 6–7 mice/treatment) and aged (n = 7–10 mice/treatment) mice. (**E,F)** Plasma glucose levels of young (**E**, n = 6–7 mice/treatment) and aged (**F**, n = 7–10 mice/treatment) mice before (basal) and during the hyperinsulinemic-euglycemic clamp (clamp) experiment. (**G,H)** Hepatic glucose production for young (**G**, n = 6–7 mice/treatment) and aged (**H**, n = 7–10 mice/treatment) mice before (basal) and during the hyperinsulinemic-euglycemic clamp (clamp) experiment. Data information: In (**B–H**), data are presented as mean ± standard deviation. For (**B,C,E–H**), an unpaired Student’s *t-*test was performed. For (**D**), 2-way ANOVA with Sidaks’s *post hoc* test was used to compare each treatment (saline *vs* rGDF11).

In order to address whether GDF11 treatment increases insulin sensitivity, we performed a 2-hour hyperinsulinemic-euglycemic clamp (2.5 mU/kg/min insulin infusion rate) in young and aged mice injected daily for 7 days with either saline or rGDF11 (Fig. 6A). Steady-state glucose infusion rates required to maintain euglycemia during the clamp were significantly higher in aged mice that received rGDF11 compared to aged mice injected with saline (Fig. 6B), indicating that rGDF11 increased systemic insulin sensitivity in aged mice. This is consistent with improved glucose tolerance in rGDF11-injected mice (Fig. S6). Insulin-stimulated whole-body glucose turnover was significantly increased in aged mice that received rGDF11 as compared to saline-treated mice (Fig. 6C). Thus, these data demonstrate that supplementation with rGDF11 increases peripheral insulin sensitivity in HFD-fed aged mice. During the clamp, a bolus of ^14^C-labeled 2-deoxyglucose was injected to assess glucose uptake in individual organs^31^. Increased peripheral insulin sensitivity was largely due to a 2-fold increase in insulin-stimulated glucose uptake in white adipose tissue, while skeletal muscle glucose metabolism was not affected by rGDF11 treatment in aged mice (Fig. S9).

Hepatic insulin resistance develops with aging and in obesity^1,32–34^. Our hyperinsulinemic-euglycemic clamp study showed that aging-associated hepatic insulin resistance was ameliorated after a 7-day dosing regimen of rGDF11. Plasma glucose levels were monitored before and during the clamp experiment in both young (Fig. 6E) and aged mice (Fig. 6F). Basal hepatic glucose production (HGP) was not affected by rGDF11 treatment in young mice (Fig. 6G,H). In contrast, clamp HGP was significantly reduced in rGDF11-injected aged mice as compared to saline-injected aged mice (Fig. 6H) which is consistent with our data showing that rGDF11 improved hepatic insulin action in aged mice (Fig. 6D). Furthermore, insulin sensitizing effects of rGDF11 were also observed in young mice, albeit less dramatic as glucose infusion rates tended to increase in young mice that received rGDF11 as compared to saline-injected young mice (Fig. 6B). Taken together, exogenous delivery of rGDF11 to aged mice resulted in an improved metabolic phenotype. However, our current approaches and data obtained to pinpoint a definitive mechanism of action suggest that there are likely multiple mechanisms responsible for the observed metabolic effects.

## Discussion

In this study, we sought to determine whether rGDF11 has specific effects on metabolism in the context of aging and HFD-induced metabolic stress by comparing the effects of exogenous rGDF11 and rGDF8 administration. Equal doses of rGDF11 or rGDF8 protein were administered daily for 11 days to young and aged mice fed either a normal chow diet, a long-term HFD, or at the commencement of a short-term HFD and the impact on multiple metabolic parameters was investigated at specific time points (Figs. 1A, 3A, and 5A). We found that supplementation with matched doses of these closely related proteins provoked different diet-dependent responses in young and aged mice.

In particular, rGDF11 treatment prevented weight gain and preserved lean mass in aged mice on a HFD, which is consistent with reductions in body weight observed in other studies following supplementation of GDF11^4,6,10^. Young and aged mice injected with rGDF11 displayed improved glucose tolerance compared to saline-injected control mice. We were surprised to find that the enhancement in glucose tolerance observed in HFD-fed aged mice following injection with rGDF11 was not associated with an increase in β-cell replication or with enhancement of fasted or glucose-stimulated insulin secretion. In addition, supplementation with rGDF11 did not improve HFD- or age-induced hepatosteatosis suggesting that the improved metabolic phenotype was not primarily driven a decline in fatty liver. Despite this, rGDF11 administration promoted weight reduction likely leading to an improved metabolic phenotype in young and aged mice fed a HFD. Together, these data suggest that supplementation with GDF11 may provide protection from HFD-induced obesity and insulin resistance, although we have been unable to isolate the exact mechanism(s) responsible for these effects. These conclusions are consistent with recent reports demonstrating that administration of rGDF11 or overexpression of GDF11 using an AAV approach promotes an improved metabolic phenotype in diabetic rodent models^8,9^.

Body weight increases when there is an imbalance in energy intake and energy expenditure, and this imbalance results in storage of excess calories^34^. In order to determine which of these parameters mediates the surprising reduction in body weight seen with GDF11 supplementation in aged mice on a HFD, we performed indirect calorimetry analysis during protein treatment. Our data suggest that rGDF11 promotes a transient reduction in food intake in aged mice as early as day 3 of treatment, but food intake was comparable to the saline injected mice by day 5. Reduced food intake was accompanied by a reduction in RER compared to saline-injected and rGDF8-injected mice, suggesting that mice injected with rGDF11 may have used their fat stores as fuel, further preventing weight gain on a HFD. The CLAMS metabolic cage analysis revealed no differences in total activity among the different treatment groups, suggesting that rGDF11 treated mice did not reduce their food intake because they were pathogenically ill, and therefore less able to move around. These observations thus raise the possibility that GDF11 may participate in appetite regulation.

A recent report suggested that GDF11 can stimulate the production of GDF15, which then acts as an appetite suppressant^14^. We tested the plasma of young and aged mice following the conclusion of the daily 0.5 mg/kg ligand dosing regimen, and we did not find any differences in circulating levels of GDF15 among the groups. This finding is consistent with another recent report showing that exogenous rGDF11 leads to a significant body weight reduction, independent of GDF15 levels, and no change in physical activity^10^. In contrast, the authors of that study did not observe a reduction in food intake, although they did not begin measuring food consumption until 3 days after the first injection^10^, the approximate time-frame when we observed a modest decrease in food intake. Those authors also observed that mice injected with rGDF11 had reduced WAT and increased serum adiponectin suggesting that rGDF11 may act to reduce body weight and WAT by stimulating adiponectin secretion^10^. Taken together, it is unlikely that the phenotypes invoked by rGDF11 are governed by a single mechanism but rather, that there are multiple mechanisms across many tissues and cell types responsible for the observed phenotypes.

Our CLAMP data revealed that glucose uptake significantly increased in WAT following exogenous delivery of rGDF11 to aged mice fed a chow diet. It is widely accepted that WAT plays a dynamic role in energy homeostasis and that WAT is highly responsive to altered energy states, both acute and chronic^34^. For example, an increase in lipid storage demand (e.g. energy abundance) can lead to insulin resistance in WAT and reduced WAT glucose uptake whereas a decrease in lipid storage demand (e.g. energy deficit) can promote insulin sensitivity and glucose uptake in WAT^34,35^. However, enhanced glucose uptake by WAT may lead to increased adiposity in the long-term, but it is also possible that the opposite may occur, where enhanced glucose uptake by WAT reduces or protects against obesity^35–40^. It is unclear how enhanced glucose uptake by WAT could promote an improved overall metabolic phenotype, but improved insulin sensitivity may facilitate glucose uptake and lead to secretion of anti-diabetic factors such as adiponectin and leptin^34,35,37^. It is possible that long term rGDF11 treatment and/or a higher dose of rGDF11 may promote reduced glucose uptake in WAT and increased obesity. In fact, acute/transient bouts vs. chronically high serum levels of non-esterified fatty acids (NEFAs) exhibit this type of short-term benefit/long-term harm paradox with regard to glucose uptake by WAT^34^. Further studies clearly will be needed to determine how (mechanism of action) and where (tissue/cell type) exogenous rGDF11 is acting to promote an improved metabolic phenotype.

Young and aged mice treated with an equal dose of rGDF8 did not exhibit many of the effects observed in rGDF11 treated mice. We found that treatment with rGDF8 or rGDF11 could prevent HFD-induced weight gain in aged mice. However, while we observed that treatment with rGDF11 prevented lean mass loss in aged mice fed a HFD, MRI measurements revealed no loss of lean mass after 7 days of rGDF8 supplementation compared to saline-injected young or aged mice on a HFD. This finding is seemingly at odds with the consensus in the field regarding the effect of elevating circulating levels of GDF8. Skeletal muscle specific overexpression of GDF8 has been shown to induce muscle atrophy and increased fat mass^19^, while disruption of *Gdf8* expression causes skeletal muscle hypertrophy and hyperplasia^16^. However, our results are consistent with experiments showing that mice that overexpress *Gdf8* transgene specifically in adipocytes are resistant to obesity on a HFD^41^. Moreover, in prior systemic supplementation studies it was reported that administration of high doses of GDF8 protein results in skeletal muscle atrophy, as well as fat mass loss, while more moderate doses of GDF8 were able to reduce fat mass without affecting skeletal muscle mass^17^. While elevated levels of GDF8 are thought to negatively impact skeletal mass, work by Zimmers and colleagues suggests that skeletal muscle atrophy following systemic GDF8 administration is dose dependent, and also that GDF8 can have selective fat mass reducing effects at lower doses^17^. In stark contrast to mice that received rGDF11, we also observed that aged mice injected with rGDF8 did not exhibit changes in food intake. Accordingly, it has been reported that neither mice with elevated levels of GDF8 nor mice with disruption of GDF8 activity show changes in food intake^20^. Mice with disrupted GDF8/GDF11/Activin signaling in muscle (dominant negative *Acvr2b* transgene) and the lipodystrophy mutation A-ZIP show reduced food intake^42^.

Previously published *Gdf8* loss of function experiments showed increased insulin sensitivity when *Gdf8* expression is disrupted^20,21^. Indeed, Wilkes and colleagues reported that systemic administration of GDF8 (0.8 μg/day) to *Gdf8* null 4 month old female mice for 5 days reduces insulin sensitivity independent of body weight^21^. Conversely, systemic administration of high and low doses of GDF8 have been shown to reduce blood glucose levels^17^ and mice that overexpress *Gdf8* specifically in adipocytes show reduced blood glucose levels and are more insulin sensitive than wild-type counterparts on both normal chow and HFD^41^. In our studies, supplementation of rGDF8 resulted in improved glucose tolerance without affecting random fed blood glucose levels in aged mice. We found that this dose of rGDF8 has an intermediate effect on glucose tolerance in aged mice under conditions of HFD compared to mice that received rGDF11. Moreover, our data indicating that aged mice injected with rGDF8 do not show improvement or exacerbation of hepatic steatosis contrasts with two studies that showed that mice with disrupted *Gdf8* expression are protected from hepatic steatosis and hepatic triglyceride accumulation^21,43^, suggesting that genetic loss of GDF8 function is not simply the opposite of gain of function by exogenous supplementation of rGDF8.

Our results are in agreement with recent reports demonstrating that supplementation with GDF11 promotes an improved metabolic phenotype in diabetic models^8,9^. Importantly, our experimental design and rGDF11 dosing regimen did not cause premature death during the experimental timeframe, did not cause anorexia, and did not result in a cachectic phenotype. It is possible that these effects may arise using a substantially-higher dose or alternative method of overexpression of GDF11^11,13–15^. We cannot exclude the possibility that using higher rGDF8 doses in the same experimental design would induce metabolic phenotypes comparable to those seen with rGDF11. Moreover, potential variations in protein potency of rGDF11 and rGDF8 may explain the different results we observed in rGDF11 *versus* rGDF8 treated mice. In support of this hypothesis, our recent data indicate that GDF11 is up to ten times more potent than GDF8 in initiating molecular signaling *in vitro* and *in vivo*^25^. Using this logic, we can hypothesize that the *in vivo* ligand-mediated effects differ due to receptor affinity/utilization or differences in their susceptibility to regulation by various binding partners. Therefore, further investigation is required to better characterize GDF11 and GDF8 receptor binding and signaling dynamics at the cellular level in order to determine the extent of possible functional differences between GDF11 and GDF8.

## Experimental Procedures

### Animal care and usage

All animal care protocols and procedures were approved by Harvard University Institutional Care and Use Committee and performed in accordance with institutional and regulatory guidelines. Unless otherwise indicated, aging C57Bl/6 male mice (24-month-old, obtained from the National Institute on Aging) and young C57Bl/6 male mice (8- to 10-week old) were housed with 12-h light/dark cycle at 22 ± 1 °C and were given *ad libitum* access to chow (5% calories from fat) and water. For studies using HFD, mice were fed chow containing 60% calories from fat (Research Diets Inc., D12492). Upon completion of experiments, mice were euthanized and organs where snap-frozen and conserved at −80 °C unless otherwise stated.

### Treatment with recombinant GDF11 or GDF8

Aged and young mice received daily intraperitoneal injection of 0.5 mg/kg of recombinant GDF11 (rGDF11), recombinant GDF8 (rGDF8) or saline (0.9% NaCl and 1 mM HCl). Recombinant proteins obtained from the manufacturer (Peprotech) were centrifuged and reconstituted in double distilled, filtered water as indicated in the datasheet. Working dilutions were prepared upon confirmation of correct protein concentration and diluted with saline solution for *in vivo* injection. Daily dosage was adjusted based on body weight changes in young and old mice.

### Glucose and insulin tolerance tests

After fasting overnight for 18 hours^44^, young and aging mice received a single intraperitoneal injection of 2 g/kg glucose to assess glucose tolerance by GTT. Blood glucose was measured instantly from nicked tails using OneTouch Ultra glucometer at 0, 15, 30, 60 and 120 min from glucose injection. Blood samples were collected at 0 and 30 min from glucose injection using Microvette CB 300 lithium-heparin coated tubes (Sarstedt) and spun 5,000 g for 5 min at 4 °C. Extracted plasma was stored at −80 °C. ITT was performed on a separate cohort of young and aging mice fasted for 5-hours. Insulin was administered at the following dosages unless otherwise noted in the figure legends: young mice fed a chow diet- 0.5 units/kg; young mice fed a HFD- 1.0 units/kg; aging mice fed a chow diet- 0.75 units/kg; aging mice fed a HFD- 1.0 units/kg. Blood glucose was measured at 0, 15, 30, 45, and 60 min as described for the GTT. Insulin and insulin c-peptide were measured by ELISA with mouse insulin standard (Alpco).

### Detection of mouse GDF15 in plasma

Plasma was collected at the conclusion of each study, aliquoted, and stored at −70C. To measure GDF15 plasma levels, stored aliquots were thawed on ice and GDF15 plasma levels were determined using the Mouse/Rat GDF15 Quantikine ELISA Kit (R&D Systems; MGD150), performed according to the manufacturer’s instructions. Samples were plated in duplicate and GDF15 concentrations were calculated using a 7-point standard curve using the provided recombinant GDF15 ligand.

### Gene expression analysis

Following tissue harvest, tissues were snap frozen in liquid nitrogen and stored at −80°c until ready for processing. RNA was extracted with Trizol reagent (Sigma), transcribed into cDNA with random primers included with the High Capacity cDNA Reverse Transcription kit (Applied Biosystems). Samples were analyzed by real-time PCR using TaqMan probes (Life Technologies). Results were normalized to expression of *Gapdh* and presented as fold increase relative to saline group for young and aged animals based on the ΔΔ*C*_*t*_ method. The catalogue numbers for the probes utilized in this study are: *Gapdh* (Mm99999915_g1), *Gdf15* (Mm00442228_m1), *Inhba* (Mm00434339_m1), *Trim63* (Mm01185221_m1), and *Fbxo32* (Mm00499523_m1).

### Indirect calorimetry, food consumption, physical activity and RER analysis

To assess multiple metabolic parameters in aging and young mice, CLAMS (Columbus Instruments) metabolic cages was employed as described by Kang and colleagues^45^. Briefly, mice were transferred to individual metabolic cages without bedding, with free access to standard chow diet and tap water and acclimated for 72 h before switching to HFD and starting 7 days of daily rGDF11, rGDF8 or saline injection. Mice were subjected to non-invasive daily monitoring of gas exchange, physical activity, and manual food weight measurements performed by a balance connected to each CLAMS metabolic cage as a readout for food intake and an Echo-MRI 3-in-1 Body composition analyzer (Echo Medical Systems) was employed to measure total body fat and lean mass. Respiratory exchange ratios (RER) were calculated as the ratio of carbon dioxide produced to oxygen consumed and energy expenditure (kcal/hour) were calculated from gas exchange. Physical activity was determined according to beam breaks within a grid of photosensors built into the cages. Total activity was defined as the total number of beam breaks, whereas ambulatory activity was determined as successive beam breaks within the grid^45^.

### Hyperinsulinemic-euglycemic clamp analysis

Young and aged mice were injected with rGDF11 or saline for 7 days and fed with HFD during the same period (Fig. 6A). Four days before hyperinsulinemic-euglycemic clamp experiments, mice were surgically implanted with a catheter in the right internal jugular vein and on the day of the clamp, a three way connector was attached to the catheter to deliver glucose and insulin^46^. After overnight fast starting the day before the clamp, a priming radioactive dose of [3-^3^H]glucose (Perkin Elmer Life and Analytical Sciences) was injected into the mice. Basal and insulin-stimulated whole-body glucose turnover was estimated with a continuous infusion of [3-^3^H]glucose during the equilibration period and throughout the clamps (0.1 μCi/min). Plasma glucose concentration was monitored during 20 min intervals and maintained at basal concentrations by infusing 20% glucose at variable rates (Fig. 6A). During the 2 h hyperinsulinemic-euglycemic clamp, mice were treated with a continuous infusion of human insulin (Humulin; Eli Lilly) at a rate of 2.5 mU/kg/min to raise plasma insulin within a physiological range. To estimate insulin-stimulated glucose uptake in individual tissues, 2-deoxy-D-[^14^C]glucose (2[^14^C]DG) (Perkin Elmer) was administered as a bolus at 120 min after the start of clamps^46^. Blood samples were taken during and at the end of clamps for measurement of plasma [3-^3^H]glucose, 2[^14^C]DG concentrations and insulin. All infusions were performed using microdialysis pumps (CMA/Microdialysis). At the end of the clamps, mice were anesthetized with sodium pentobarbital injection and skeletal muscles, epididymal white adipose tissue, and liver were harvested for analysis. Biochemical assays to analyze glucose turnover and glucose uptake were performed as described in^46^.

### Immunofluorescence and histology

For hepatosteatosis analysis, freshly dissected livers were fixed with 4% (w/v) paraformaldehyde for 24 h at 4 °C and paraffin embedded the following day. Five μm sections were deparaffinized in xylene, rehydrated by graded alcohols, stained with iron hematoxylin for 3 min and then rinsed in deionized water for 2 min. After two quick dips in Acid Alcohol, sections were washed 2 min in deionized water, submerged in Scott’s blueing agent for 3 min, rinsed in deionized water and stained with eosin for 2 min. After a final step of xylene for 2 min, slides were mounted with a coverslip. Dried slides were used for quantification of hepatosteatosis uning a 40X magnification on a Olympus BX41 microscope and, for each mouse, at least ten nonconsecutive random digital images were obtained. Severity of steatosis was defined according to the percentage of affected hepatocytes: 0 = <5%, 1 = 5–33%, 2 = 34–66%, 3 = ≥67% as described in^47^. For pancreatic β-cell replication, freshly disected pancreata were fixed with 4% (w/v) paraformaldehyde for 24 h at 4 °C. The pancreata were processed for cryoperservation using 30% (w/v) sucrose for 24 h at 4 °C and subsequently embedded in OCT and frozen and stored at −80 °C. OCT embedded pancreata were sectioned every 200 μm generating at least 9 sections. Sections were stained using primary antisera including guinea pig anti-insulin (#A0564; Dako, Carpinteria, CA), mouse anti-Nkx6.1 (Developmental Studies Hybridoma Bank, Iowa City, Iowa) and rabbit anti-Ki67 SP6 (#ab16667; Abcam, Cambridge, MA) along with donkey secondary antisera conjugated to Cy2, Cy3, or Cy5 (#A21206, #A21203, A#31571; LifeTechnology, Carlsbad, CA).

### Proliferation analysis

Every islet in 6–8 sections across two slides were imaging using the 20x objective and the 10x eyepiece of the Zeiss Imager Z2 microscope for quantification of β-cell proliferation. Ki67^+^ insulin^+^ DAPI^+^ cells were counted manually using the multi-point tool of the ImageJ software. Ki67^+^ β-cell ratios were calculated as percent of total insulin positive cells.

### Statistical analysis

Statistical comparison was performed by one-way ANOVA or two-way ANOVA with *post hoc* multiple comparison analysis as indicated. One-way ANOVA was used to assess the significance of differences observed across the three treatment groups on a single variable. Two-way ANOVA was used to assess the effect of two factors (e.g. time and treatment type) on a measurable variable. Statistical analysis was carried out with the Graphpad Prism 7 Software and statistical significance was assigned to differences with a *p* value < 0.05.

## Supplementary information

Supplementary information.

## References

[1] Fink RI, Kolterman OG, Griffin J, Olefsky JM (1983). Mechanisms of insulin resistance in aging. J. Clin. Invest..

[2] Kushner JA (2013). The role of aging upon β cell turnover. J. Clin. Invest..

[3] Katsimpardi L (2014). Vascular and neurogenic rejuvenation of the aging mouse brain by young systemic factors. Science.

[4] Ozek C, Krolewski RC, Buchanan SM, Rubin LL (2018). Growth Differentiation Factor 11 treatment leads to neuronal and vascular improvements in the hippocampus of aged mice. Sci. Rep..

[5] Sinha M (2014). Restoring systemic GDF11 levels reverses age-related dysfunction in mouse skeletal muscle. Science.

[6] Loffredo FS (2013). Growth differentiation factor 11 is a circulating factor that reverses age-related cardiac hypertrophy. Cell.

[7] Poggioli T (2015). Circulating Growth Differentiation Factor 11/8 Levels Decline with Age. Circulation Research.

[8] Li H (2017). GDF11 Attenuates Development of Type 2 Diabetes via Improvement of Islet β cell Function and Survival. Diabetes.

[9] Zhang Jiajia, Li Yixiang, Li Huan, Zhu Biao, Wang Li, Guo Bei, Xiang Lin, Dong Jing, Liu Min, Xiang Guangda (2018). GDF11 Improves Angiogenic Function of EPCs in Diabetic Limb Ischemia. Diabetes.

[10] Katsimpardi L (2019). Systemic GDF11 stimulates the secretion of adiponectin and induces a calorie restriction-like phenotype in aged mice. Aging Cell.

[11] Egerman MA (2015). GDF11 Increases with Age and Inhibits Skeletal Muscle Regeneration. Cell Metabolism.

[12] Schafer MJ (2016). Quantification of GDF11 and Myostatin in Human Aging and Cardiovascular Disease. Cell Metabolism.

[13] Hammers DW (2017). Supraphysiological levels of GDF11 induce striated muscle atrophy. EMBO Molecular Medicine.

[14] Jones JE (2018). Supraphysiologic Administration of GDF11 Induces Cachexia in Part by Upregulating GDF15. Cell Reports.

[15] Harper SC (2018). GDF11 Decreases Pressure Overload-Induced Hypertrophy, but Can Cause Severe Cachexia and Premature Death. Circulation Research.

[16] McPherron AC, Lee S-J (1997). Regulation of skeletal muscle mass in mice by a new TGF-beta superfamily member. Nature.

[17] Zimmers TA (2002). Induction of cachexia in mice by systemically administered myostatin. Science.

[18] McPherron AC, Lee S-J (1997). Double muscling in cattle due to mutations in the myostatin gene. Proceedings of the National Academy of Sciences.

[19] Reisz-Porszasz S (2003). Lower skeletal muscle mass in male transgenic mice with muscle-specific overexpression of myostatin. AJP: Endocrinology and Metabolism.

[20] McPherron AC, Lee S-J (2002). Suppression of body fat accumulation in myostatin-deficient mice. J. Clin. Invest..

[21] Wilkes JJ, Lloyd DJ, Gekakis N (2009). Loss-of-function mutation in myostatin reduces tumor necrosis factor alpha production and protects liver against obesity-induced insulin resistance. Diabetes.

[22] Lee S-J (2010). Extracellular Regulation of Myostatin: A Molecular Rheostat for Muscle Mass. IEMAMC.

[23] McPherron AC (2010). Metabolic Functions of Myostatin and GDF11. Immunol Endocr Metab Agents Med Chem.

[24] Walker RG (2016). Biochemistry and Biology of GDF11 and Myostatin Similarities, Differences, and Questions for Future Investigation. Circulation Research.

[25] Walker RG (2017). Structural basis for potency differences between GDF8 and GDF11. BMC Biol.

[26] Barzilai N, Rossetti L (1995). Relationship between changes in body composition and insulin responsiveness in models of the aging rat. The American Journal of Physiology.

[27] Harmon EB (2004). GDF11 modulates NGN3+ islet progenitor cell number and promotes β-cell differentiation in pancreas development. Development.

[28] Dichmann DS, Yassin H, Serup P (2006). Analysis of pancreatic endocrine development in GDF11‐deficient mice. Developmental Dynamics.

[29] Hansen BC (1999). The Metabolic Syndrome X. Annals of the New York Academy of Sciences.

[30] Barzilai N, Rossetti L (1996). Age-related changes in body composition are associated with hepatic insulin resistance in conscious rats. The American Journal of Physiology.

[31] Rankin MM, Kushner JA (2009). Adaptive beta-cell proliferation is severely restricted with advanced age. Diabetes.

[32] Jackson RA (1988). Influence of aging on hepatic and peripheral glucose metabolism in humans. Diabetes.

[33] Kahn BB, Flier JS (2000). Obesity and insulin resistance. J. Clin. Invest..

[34] Rosen ED, Spiegelman BM (2006). Adipocytes as regulators of energy balance and glucose homeostasis. Nature.

[35] Smith U, Kahn BB (2016). Adipose tissue regulates insulin sensitivity: role of adipogenesis, de novo lipogenesis and novel lipids. J. Intern. Med..

[36] Terauchi Y (2004). Increased serum leptin protects from adiposity despite the increased glucose uptake in white adipose tissue in mice lacking p85alpha phosphoinositide 3-kinase. Diabetes.

[37] Muñoz S (2010). Chronically increased glucose uptake by adipose tissue leads to lactate production and improved insulin sensitivity rather than obesity in the mouse. Diabetologia.

[38] Raciti GA, Bera TK, Gavrilova O, Pastan I (2011). Partial inactivation of Ankrd26 causes diabetes with enhanced insulin responsiveness of adipose tissue in mice. Diabetologia.

[39] Elias I, Franckhauser S, Bosch F (2013). New insights into adipose tissue VEGF-A actions in the control of obesity and insulin resistance. Adipocyte.

[40] Elias I (2012). Adipose tissue overexpression of vascular endothelial growth factor protects against diet-induced obesity and insulin resistance. Diabetes.

[41] Feldman BJ, Streeper RS, Farese RV, Yamamoto KR (2006). Myostatin modulates adipogenesis to generate adipocytes with favorable metabolic effects. Proceedings of the National Academy of Sciences.

[42] Guo T (2012). Myostatin inhibition prevents diabetes and hyperphagia in a mouse model of lipodystrophy. Diabetes.

[43] Guo T (2009). Myostatin inhibition in muscle, but not adipose tissue, decreases fat mass and improves insulin sensitivity. Plos One.

[44] Ayala JE (2010). Standard operating procedures for describing and performing metabolic tests of glucose homeostasis in mice. Disease Models & Mechanisms.

[45] Kang HW (2013). Thioesterase superfamily member 2/Acyl-CoA thioesterase 13 (Them2/Acot13) regulates adaptive thermogenesis in mice. J. Biol. Chem..

[46] Kim JK (2004). Inactivation of fatty acid transport protein 1 prevents fat-induced insulin resistance in skeletal muscle. J. Clin. Invest..

[47] Hübscher SG (2006). Histological assessment of non-alcoholic fatty liver disease. Histopathology.

