# Supplementary material for: Exogenous GDF11, but not GDF8, reduces body weight and improves glucose homeostasis in mice
Source: Sci Rep. 2020 Mar 12;10:4561. doi: 10.1038/s41598-020-61443-y (PMC7067781; doi:10.1038/s41598-020-61443-y)
Supplement: Supplementary file 1 — Supplementary information. [file 41598_2020_61443_MOESM1_ESM.docx]

Exogenous GDF11, but not GDF8, reduces body weight and improves glucose homeostasis in mice

Ryan G. Walker^1#^, Ornella Barrandon^1#^, Tommaso Poggioli^1^, Sezin Dagdeviren^1^, Shannon H. Carroll^1^, Melanie J. Mills^1^, Kourtney R. Mendello^1^, Yanet Gomez^1^, Francesco S. Loffredo^1^, James R. Pancoast^1^, Claudio Macias-Trevino^1^, Colin Marts^1^, Katherine B. LeClair^2^, Hye-Lim Noh^3^, Taekyoon Kim^3^, Alexander S. Banks^4^, Jason K. Kim^3^, David E. Cohen^5^, Amy J. Wagers^1,6^, Douglas A. Melton^1^, Richard T. Lee^1*^

^#^Co-first author

From the ^1^Department of Stem Cell and Regenerative Biology, Harvard University, Cambridge, MA 02138, USA; ^2^Division of Gastroenterology, Hepatology and Endoscopy, Brigham and Women’s Hospital and Harvard Medical School, Boston, MA 02139, USA; ^3^Program in Molecular Medicine, University of Massachusetts Medical School, Worcester, MA 01605, USA; ^4^Division of Endocrinology, Diabetes and Metabolism, Beth Israel Deaconess Medical Center and Harvard Medical School, Boston, MA; ^5^Division of Gastroenterology and Hepatology, Weill Cornell Medicine, New York, NY, USA; ^6^Paul F. Glenn Center for the Biology of Aging, Harvard Medical School, Boston, MA 02115.

Running title: *GDF11 improves glucose homeostasis*

**Supporting Information Contents**

**Figure S1**

**Figure S2**

**Figure S3**

**Figure S4**

**Figure S5**

**Figure S6**

**Figure S7**

**Figure S8**

**Figure S9**


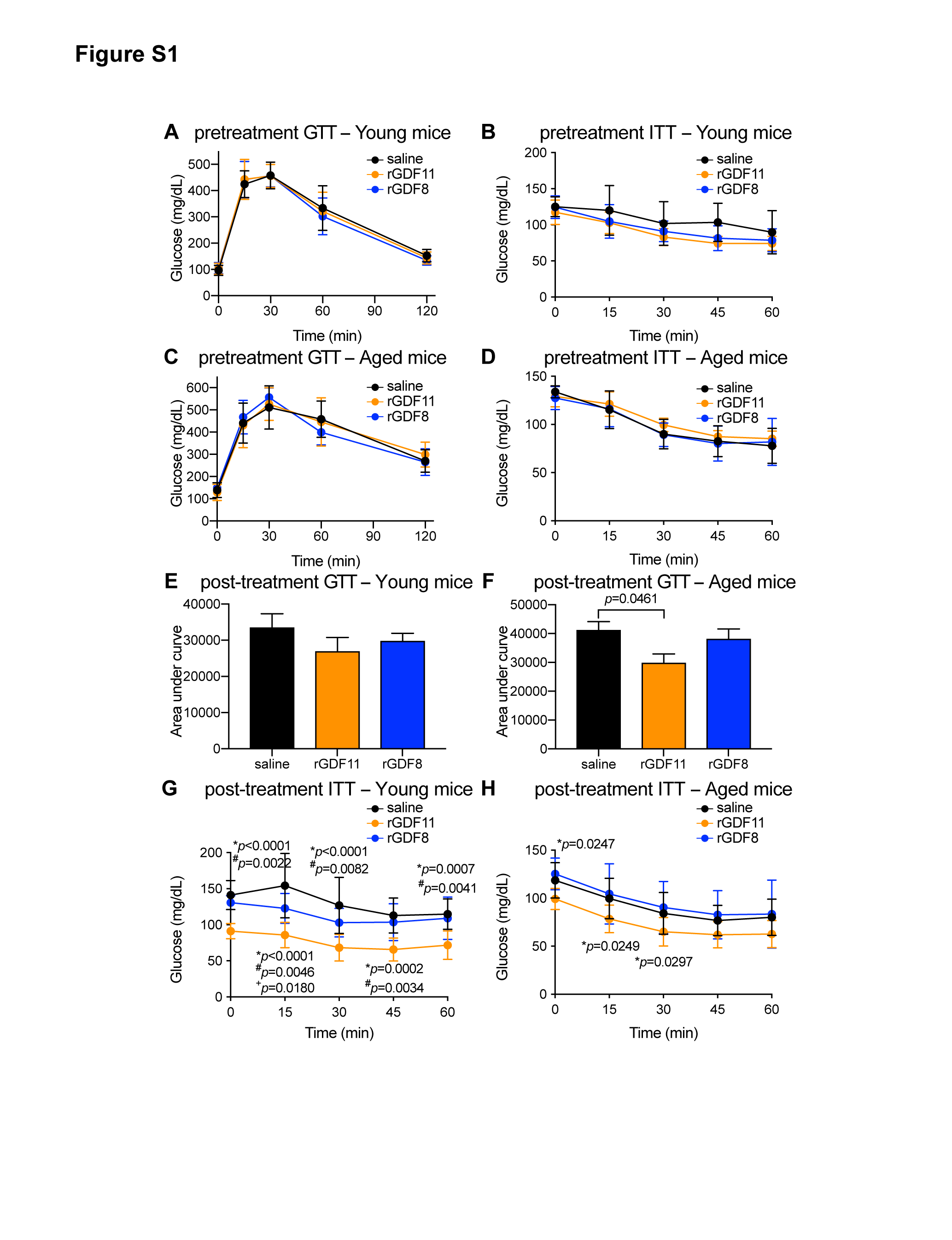


**Figure S1 - rGDF11, but not rGDF8, improved glucose tolerance in aged mice.**

**A, B** Baseline GTT (**A**, n=6 mice/treatment) and ITT (**B**, n=9 mice/treatment) for young mice fed a normal chow diet. For GTT, mice were fasted for 18 hours overnight before a bolus of glucose was administered (2g/kg). For ITT, mice were fasted for 5 hours before injected with 0.5 units/kg of insulin.

**C, D** Baseline GTT (**C**, n=6-8 mice/treatment) and ITT (**D**, n=9 mice/treatment) for aged mice fed a normal chow diet. For GTT, mice were fasted for 18 hours overnight before a bolus of glucose was administered (2g/kg). For ITT, mice were fasted for 5 hours before injected with 0.75 units/kg of insulin.

**E, F** Calculated area under the curve (AUC) corresponding to **Fig 1** GTT for young and aged mice fed a normal chow diet (n=6/mice treatment).

**G, H** Raw glucose values measured for data presented in **Fig 1F** and **Fig 1G**, respectively (n=9 mice/treatment group).

Data information: In (**A-D, G, H**), data for the GTT and ITT are presented as mean ± standard deviation. (**E, F**) The AUC data is presented as mean ± standard error of the mean. For (**A-D, G, H**), GTT and ITT data was assessed using 2-way ANOVA with Tukey’s *post hoc* test for comparison between groups. For AUC data in (**E, F**), 1-way ANOVA with Tukey’s *post hoc* test was used. For all graphs: * - saline *vs*. rGDF11; ^#^ - rGDF11 *vs.* rGDF8; ^+^ - saline *vs.* rGDF8.

**
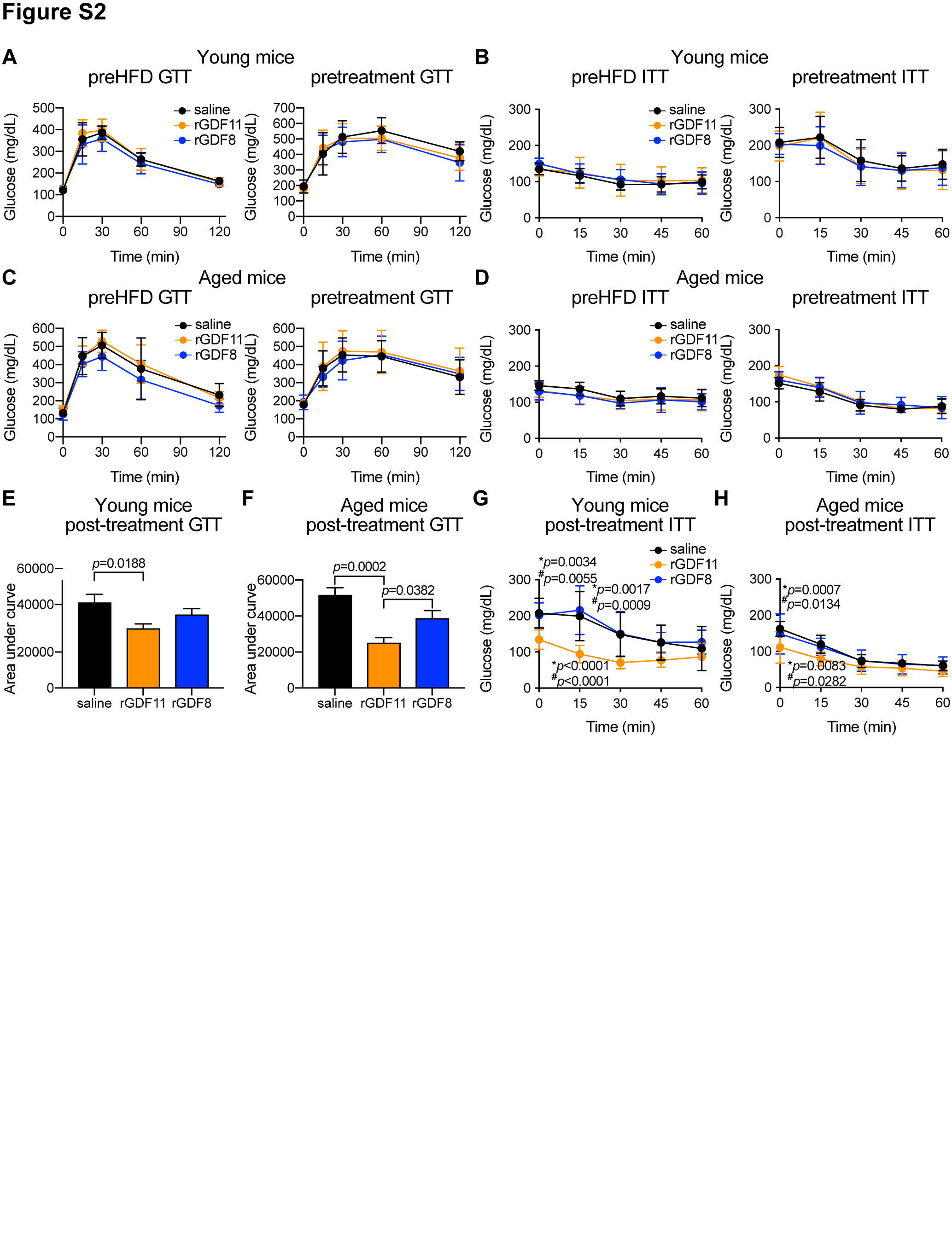
**

**Figure S2 – Glucose tolerance and insulin tolerance tests preHFD, postHFD, and post rGDF11 or rGDF8 administration.**

**A, B** Baseline GTT (**A**, n=7-8 mice/treatment) and ITT (**B**, n=8-9 mice/treatment) for young mice fed a HFD. ‘preHFD’ is defined as before starting mice on 8-week HFD and ‘pretreatment’ is defined as before exogenous administration of saline, rGDF11, or rGDF8. For GTT, mice were fasted for 18 hours overnight before a bolus of glucose was administered (2g/kg). For ITT, mice were fasted for 5 hours before injected with insulin (preHFD ITT, 0.5 units/kg insulin; pretreatment ITT, 1.0 units/kg of insulin).

**C, D** Baseline GTT (**C**, n=6-8 mice/treatment) and ITT (**D**, n=7-10 mice/treatment) for aged mice fed a HFD. For GTT, mice were fasted for 18 hours overnight before a bolus of glucose was administered (2g/kg). For ITT, mice were fasted for 5 hours before injected with insulin (preHFD ITT, 0.75 units/kg insulin; pretreatment ITT, 1.0 units/kg of insulin).

**E, F** Calculated AUC corresponding to **Fig** **2** GTT for young (**E**, n=7-8 mice/treatment) and aged (**F**, n=6-8 mice/treatment) fed a HFD.

**G, H** Raw glucose values measured for data presented in **Fig 2F** (**G**, n=8-9 mice/treatment) and **Fig 2G** (**H**, n=7-10 mice/treatment), respectively.

Data information: In (**A-D, G, H**), data for the GTT and ITT are presented as mean ± standard deviation. For (**E, F**), the AUC data is presented as mean ± standard error of the mean. For (**A-D, G, H**), GTT and ITT data was assessed using 2-way ANOVA with Tukey’s *post hoc* test for comparison between groups. For AUC data in (**E, F**), 1-way ANOVA with Tukey’s *post hoc* test was used. For all graphs: * - saline *vs*. rGDF11; ^#^ - rGDF11 *vs.* rGDF8.

**
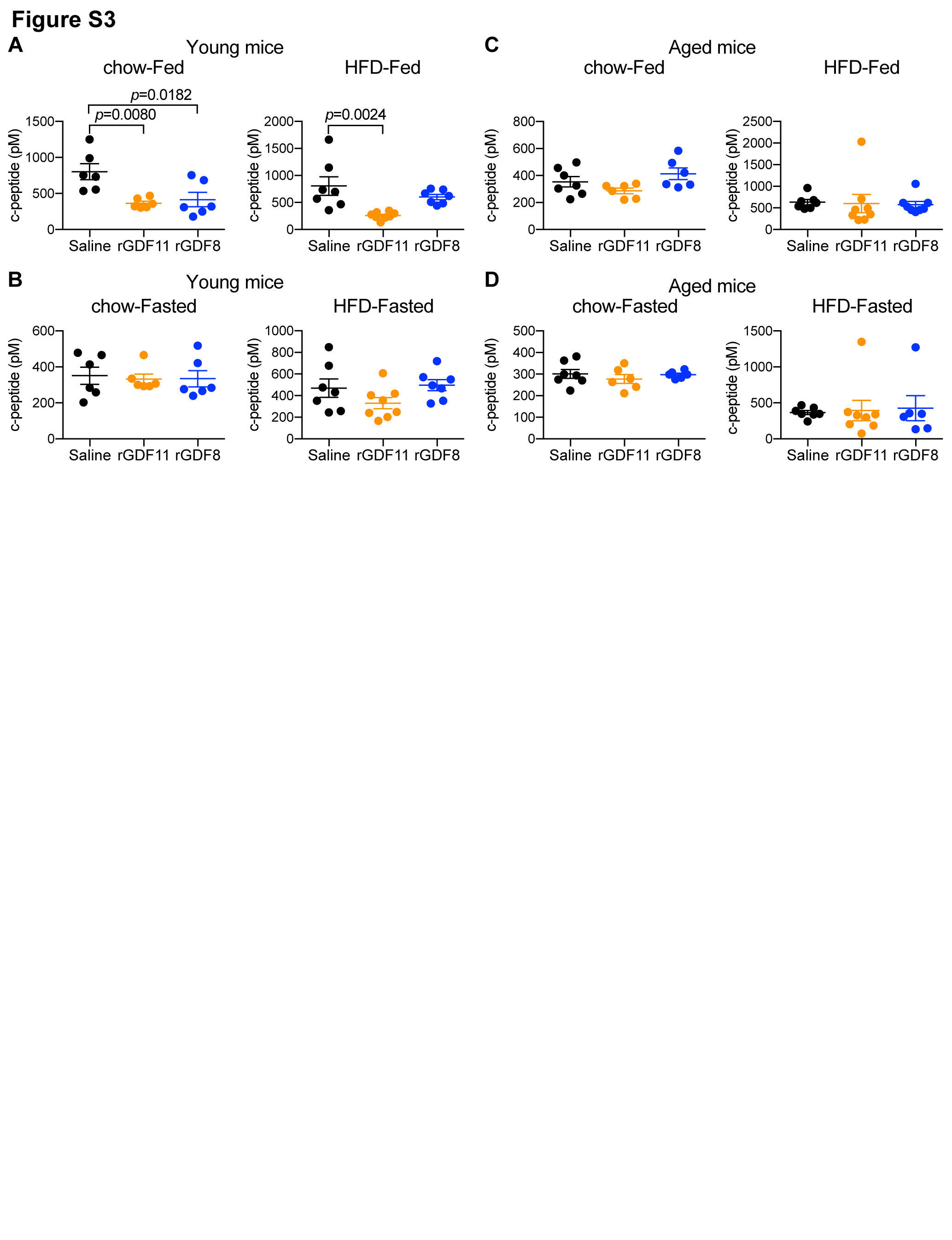
**

**Figure S3 - Fed and fasting insulin c-peptide measurements in young and aged mice fed a normal chow diet or high fat diet following administration of saline, rGDF11, or rGDF8.**

**A** Fed c-peptide levels in young mice fed a normal chow diet (left, n=6 mice/treatment) and HFD (right, n=7-8 mice/treatment).

**B** Fasting (18 hours) insulin c-peptide levels in young mice fed a normal chow diet (left, n=6 mice/treatment) and HFD (right, n=7-8 mice/treatment).

**C** Fed insulin c-peptide levels in aged mice fed a normal chow diet (left, n=6-8 mice/treatment) and HFD (right, n=6-8 mice/treatment).

**D** Fasting (18 hours) insulin c-peptide levels in aged mice fed a normal chow diet (left, n=6 mice/treatment) and HFD (right, n=6-8 mice/treatment).

Data information: In (**A-D**), data is presented as mean ± standard error of the mean and1-way ANOVA with Tukey’s *post hoc* test was used to assess significance.


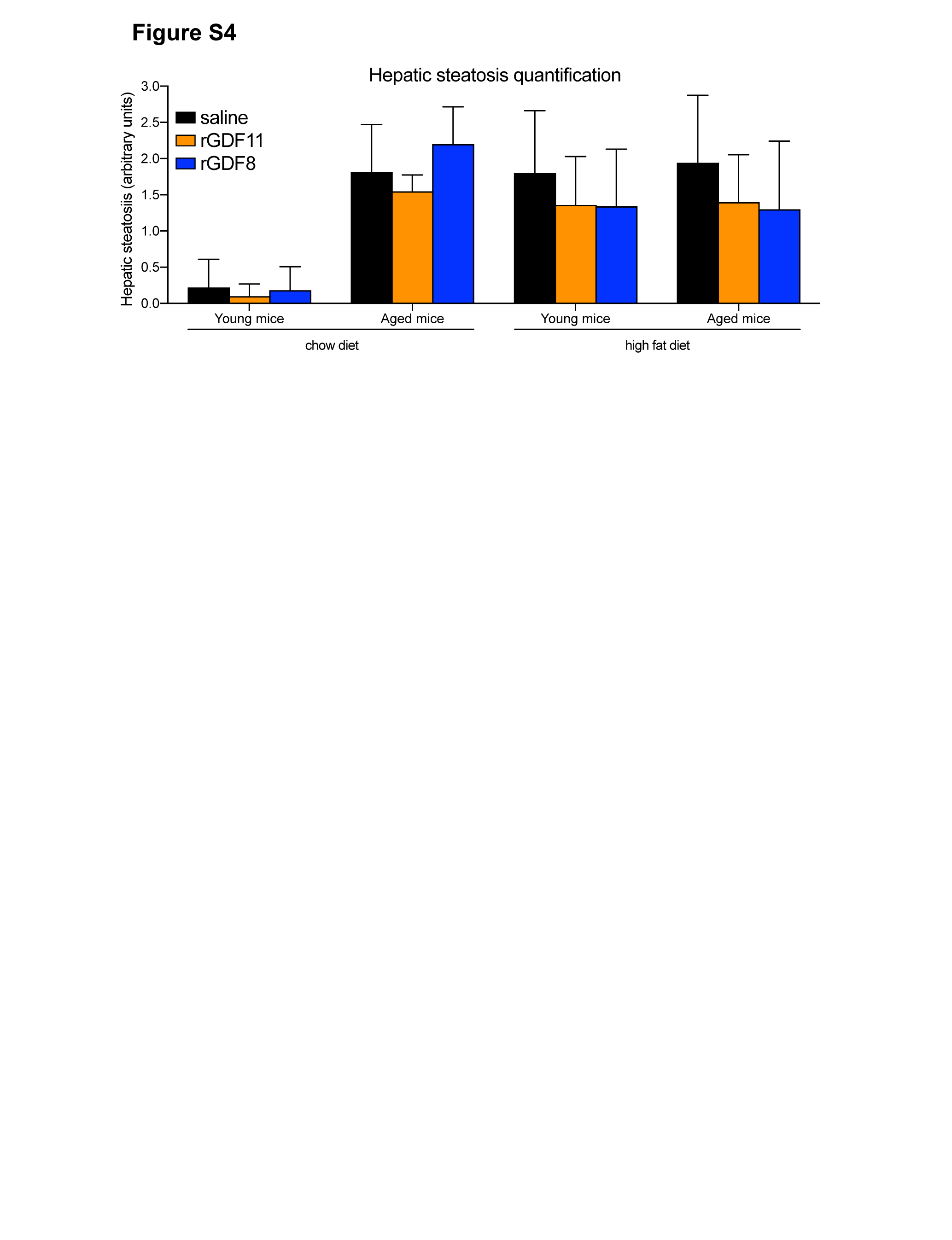


**Figure S4 - No improvement in hepatic morphology in young or aged mice following exogenous delivery of rGDF11 or rGDF8 fed a normal chow diet or long-term HFD.**

Quantification of liver sections from young mice on a normal chow diet (n=5-6 mice/treatment) or long-term HFD (n=7-8 mice/treatment) and aged mice on a normal chow diet (n=6-7 mice/treatment) or long-term HFD (n=6-8 mice/treatment) following daily injection of saline (black), rGDF11 (orange), or rGDF8 (blue) for 11 days.

Data information: Data are presented as mean ± standard deviation. A 2-way ANOVA with either Sidak’s or Tukey’s *post hoc* test was used for comparison between young *vs.* aged treatment or comparison between groups, respectively.


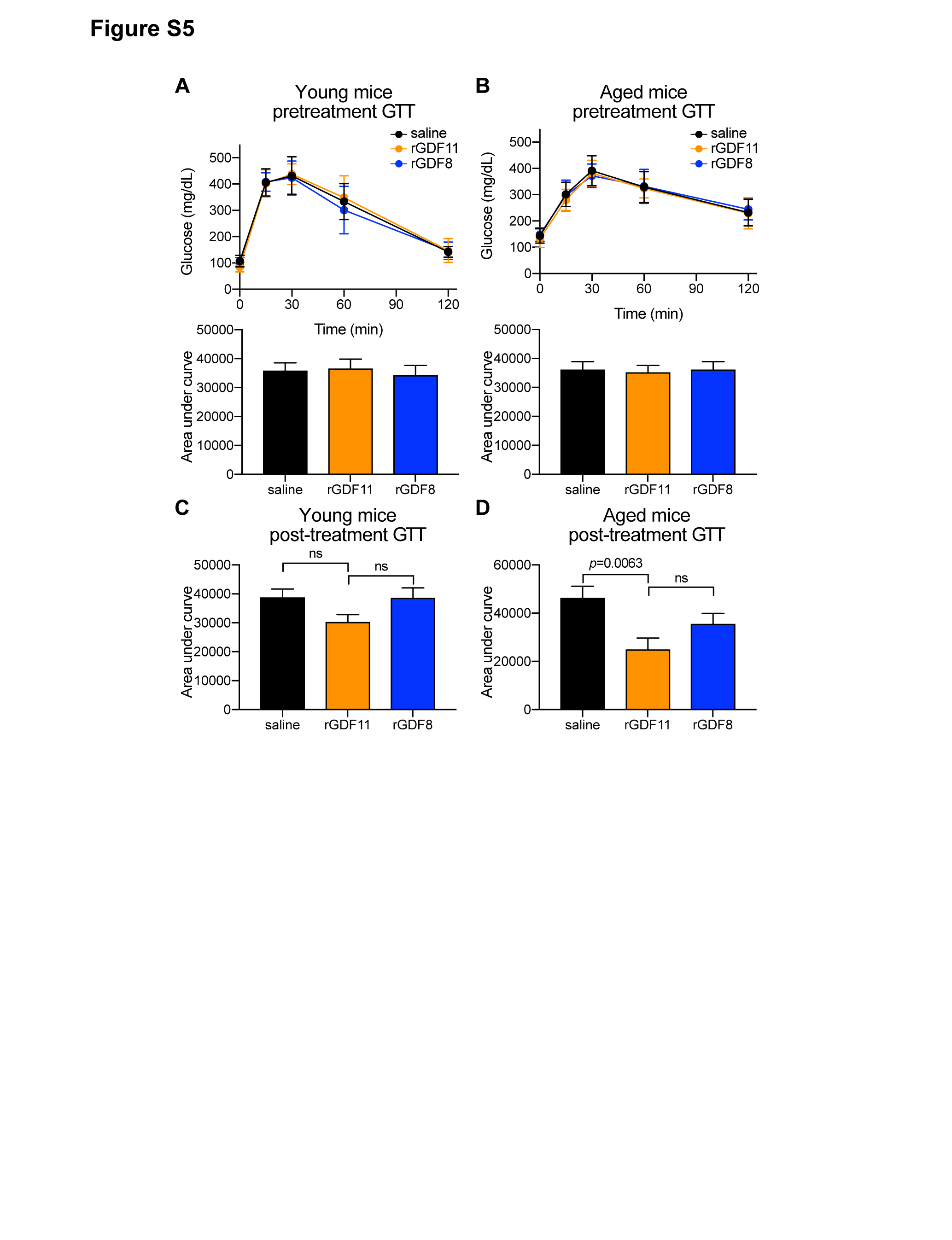


**Figure S5 – Pre-and post-treatment glucose tolerance and insulin tolerance tests from mice fed a short-term HFD at the onset rGDF11 or rGDF8 administration.**

**A, B** Baseline GTT for young (**A**, n=11-12 mice/treatment) and aged mice fed a HFD (**B**, n=7-8 mice/treatment). ‘pretreatment’ is defined as before exogenous administration of saline, rGDF11, or rGDF8. The area under the curve (AUC) is shown below each graph. For GTT, mice were fasted for 18 hours overnight before a bolus of glucose was administered (2g/kg).

**C, D** Calculated AUC corresponding to **Fig** **5** GTT for young (**C**, n=11-12 mice/treatment) and aged (**D**, n=11-12 mice/treatment) fed a short-term HFD.

Data information: In (A, B), data for the GTT are presented as mean ± standard deviation. In (A-D), the AUC data are presented as mean ± standard error of the mean. For (A-D), GTT data was assessed using 2-way ANOVA with Tukey’s *post hoc* test for comparison between groups. For AUC data in (A-D), 1-way ANOVA with Tukey’s *post hoc* test was used.


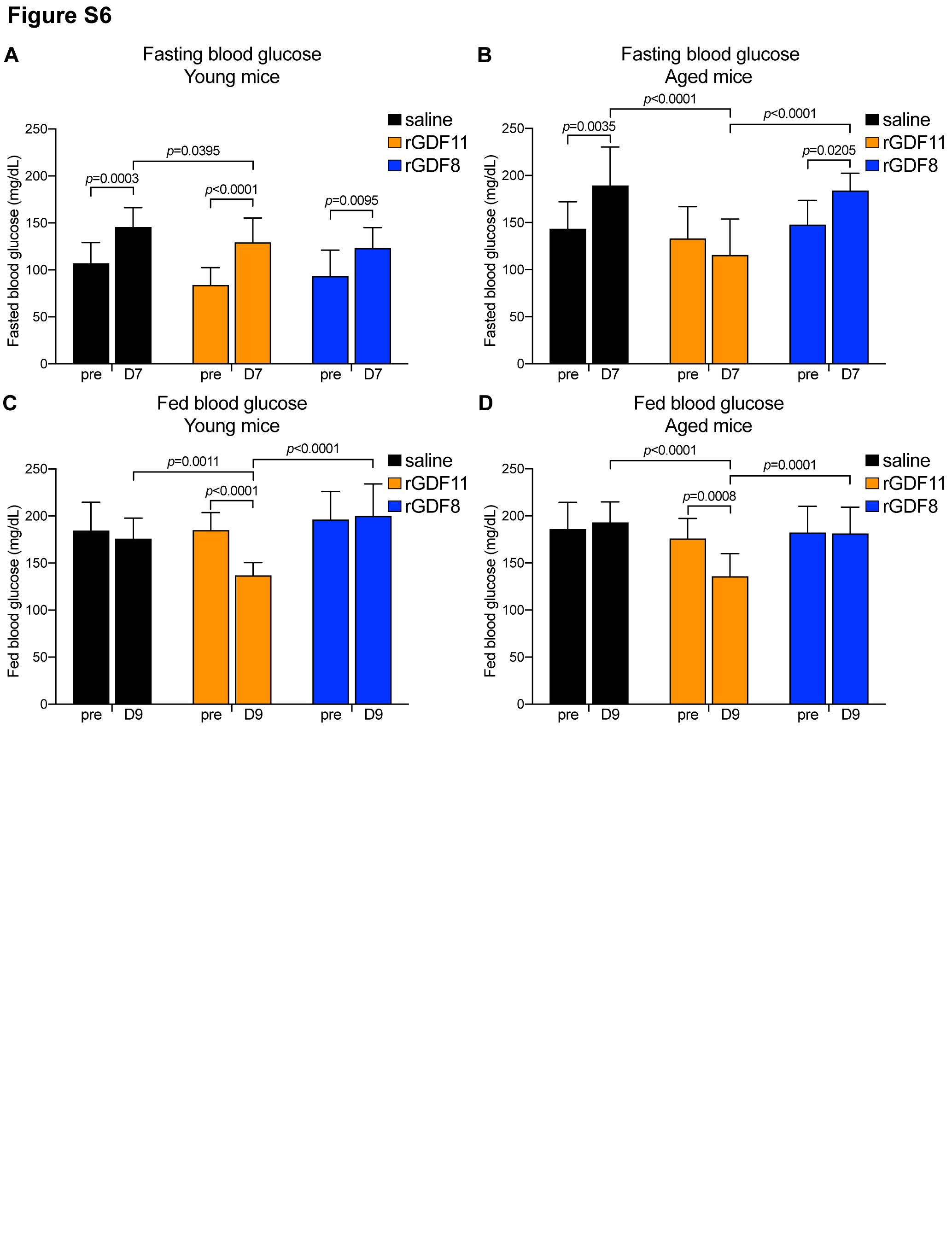


**Figure S6 - Exogeneous rGDF11, but not rGDF8, prevents HFD-induced elevation in blood glucose levels.**

**A, B** Blood glucose levels for young (**A**, n=11-12 mice/treatment) and aged (**B**, n=11-12 mice/treatment) mice were fasted 18 hours overnight and fasted blood glucose levels were measured prior to (Pre) and after 7 days (D7) of a short-term HFD feeding and saline (black), rGDF11 (orange), or rGDF8 (blue) treatment.

**C, D** Random fed blood glucose levels were measured from young (**C**, n=11-12 mice/treatment) and aged (**D**, n=11-12 mice/treatment) mice prior to (Pre) and after 9 days (D9) of a short-term HFD feeding and saline (black), rGDF11 (orange), or rGDF8 (blue) treatment.

Data information: In (**A-D**), data are presented as mean ± standard deviation. For (**A-D**), 2-way ANOVA with either Sidak’s or Tukey’s *post hoc* test was used for comparison between pre *vs.* post treatment or comparison between groups, respectively.


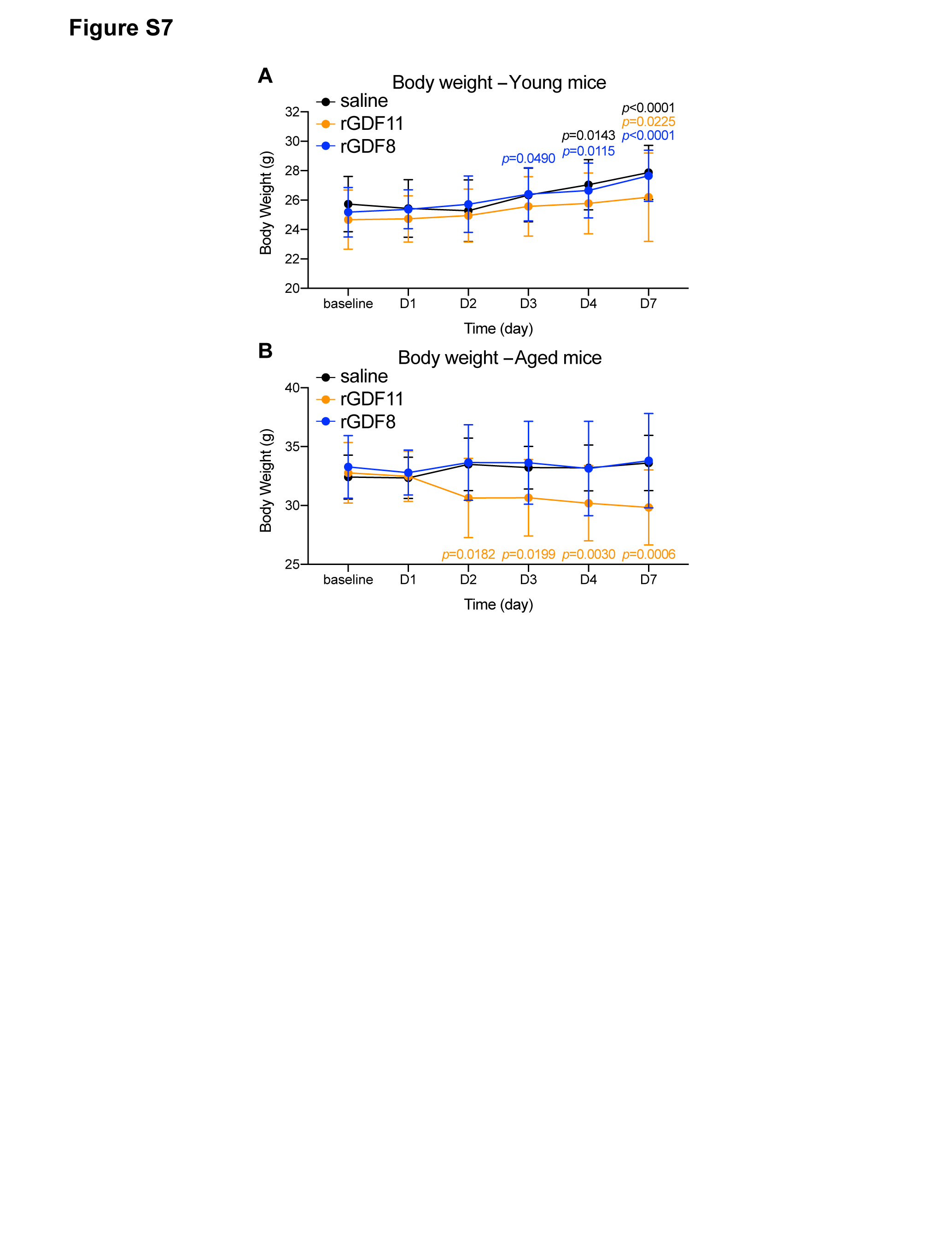


**Figure S7 - Aged mice fed a short-term HFD with concomitant administration of exogenous rGDF11, but not of rGDF8, show reduction in body weight.**

**A, B** Body weights measure daily of young (**A**, n=4-6 mice/treatment) and aged (**B**, n=5-6 mice/treatment) mice during concomittant HFD feeding and administration of saline (black), rGDF11 (orange), or rGDF8 (blue). Statistical signicance values are color matched and reported as a comparison of each data point to initial baseline values.

Data information: In (**A, B**), data are presented as mean ± standard deviation. For (**A, B**), 2-way ANOVA with Dunnett’s *post hoc* test was used to compare each treatment to the resepctive baseline.


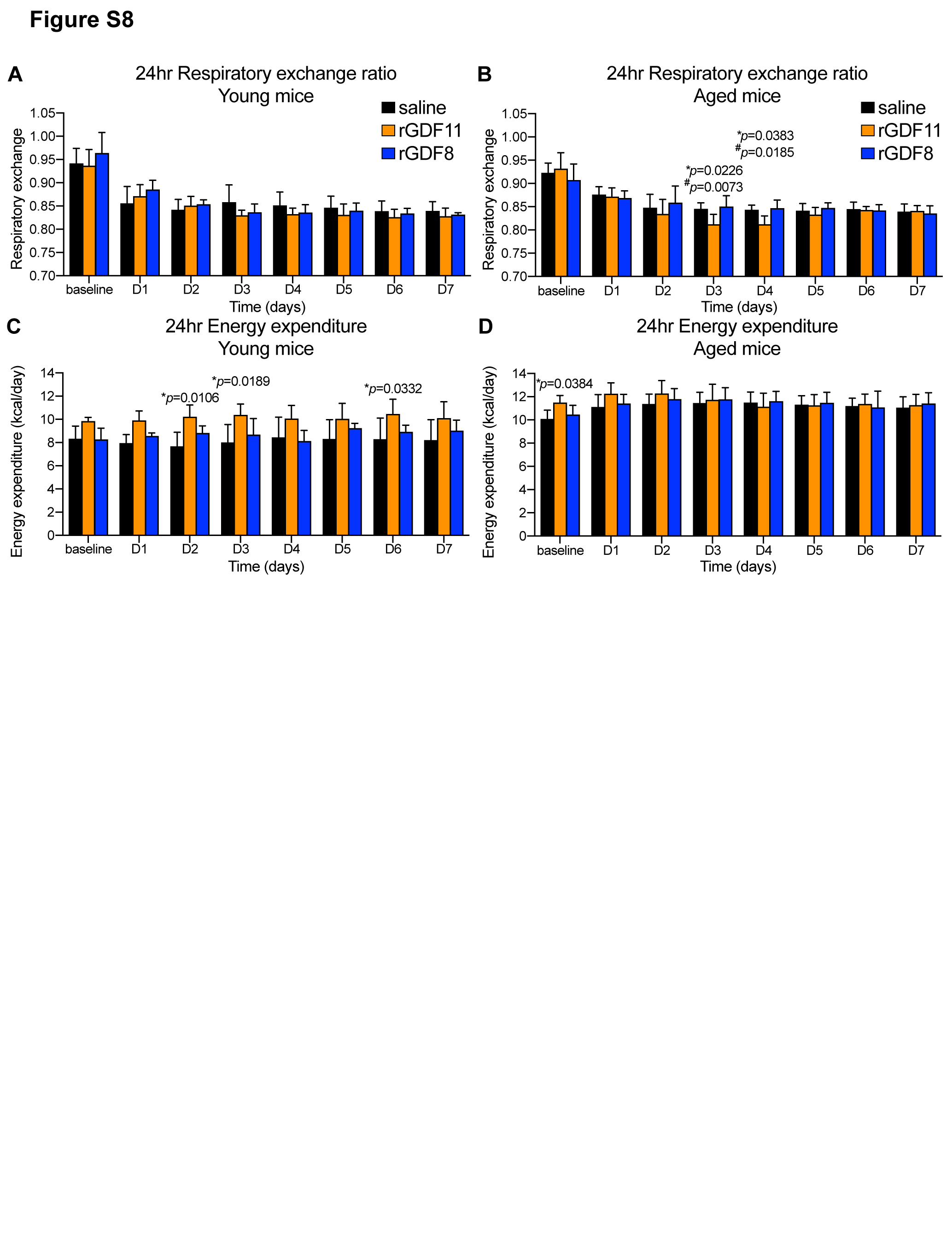


**Figure S8 - Comprehensive Laboratory Animal Monitoring System (CLAMS) analysis reveals that aged mice fed a short-term HFD with concomitant administration of exogenous rGDF11, but not of rGDF8, show a transient reduction in RER.**

**A-D** Respiratory exchange ratio (RER; **A, B**) and energy expenditure (**C, D**) were measured from young (**A, C**, n=4-6 mice/treatment) and aged (**B, D**,n=5-6 mice/treatment). Prior to concomittant short-term HFD feeding and delivery of either saline, rGDF11, or rGDF8, baseline values were established (baseline) and then monitored daily during CLAMS analysis.

Data information: In (**A-D**), data are presented as mean ± standard deviation. For (**A-D**), 2-way ANOVA with Tukeys’s *post hoc* test was used to compare each treatment group on the specific day.


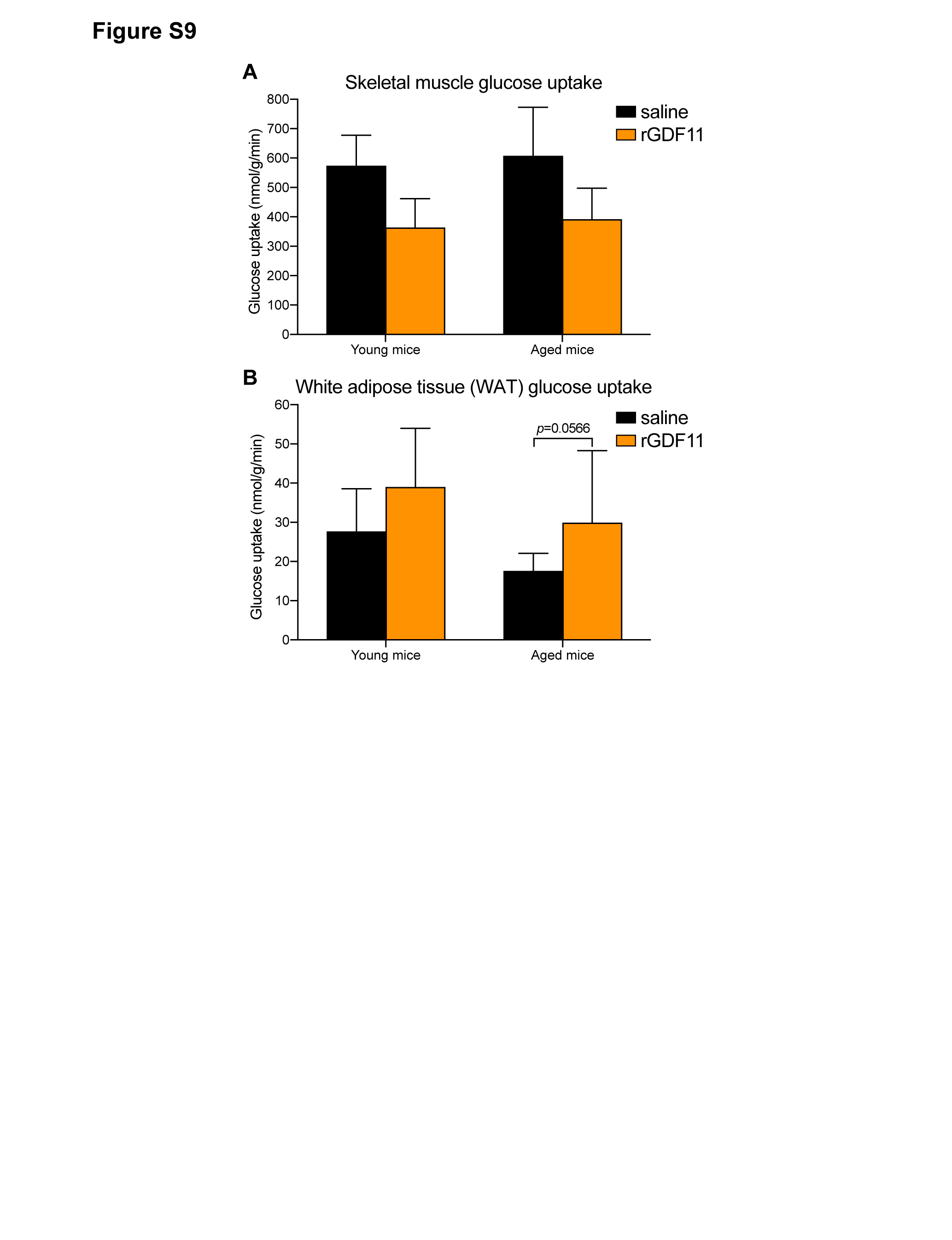


**Figure S9 - Young and aged mice fed a short-term HFD with concomitant administration of exogenous rGDF11 ddoes not increase in peripheral glucose uptake.**

**A, B** Skeletal muscle (**A**) and white adipose tissue (WAT; **B**) glucose uptake in young (n=6-7 mice/treatment) and aged (n=7-10 mice/treatment) during the hyperinsulinemic-euglycemic clamp experiment on mice fed a short-term HFD and simultaneosuly received a daily injection of either saline (black) or rGDF11 (orange) for 1 week.

Data information: In (**A, B**), data are presented as mean ± standard deviation. For (**A, B**), an unpaired Student’s *t-*test was performed.
